# Surface-driven size scaling of the lattice parameter, Debye–Waller coefficient and microstrain in nanocrystals

**DOI:** 10.1107/S2053273326007345

**Published:** 2026-08-03

**Authors:** Sirisha Subbareddy, Erick Agnolin, Marcelo Augusto Malagutti, Narges Ataollahi, Robert Dinnebier, Paolo Scardi

**Affiliations:** a Department of Civil, Environmental and Mechanical Engineering University of Trento via Mesiano 77 Trento 38123 Italy; b Max Planck Institute for Solid State Research Heisenbergstrasse 1 Stuttgart 70569 Germany; Institute of Crystallography - CNR, Bari, Italy

**Keywords:** nanocrystals, lattice parameter, Debye–Waller coefficient, microstrain

## Abstract

Nanocrystals exhibit size-dependent structural behaviour because surface and interface effects become increasingly important as the characteristic domain size decreases. In this work, we investigate the size dependence of three diffraction-derived structural parameters – lattice parameter, isotropic Debye–Waller coefficient and microstrain – by combining molecular dynamics simulations of spherical Pd, Fe and Ti nanocrystals with powder diffraction analysis and comparison with representative literature data. The atomistic results show that small nanocrystals are characterized by a mean compressive state together with pronounced surface-stress inhomogeneity, while enhanced atomic displacements are concentrated in the outer coordination shells. On this basis, three simple nanoscale hypotheses are proposed. The lattice parameter is described as an ideal capillarity-driven reference trend with leading 1/*D* (where *D* is the particle diameter) behaviour, although comparison with experimental data confirms that this quantity is not universal and may be strongly modified by surface chemistry, defects, non-stoichiometry and morphology. By contrast, the Debye–Waller coefficient follows a more general 1/*D* decrease, consistent with a surface-shell picture of enhanced vibrational and static disorder. Microstrain arises from surface-stress gradients and, in the small-particle limit, exhibits a natural 1/*D*^2^ dependence associated with surface-stress heterogeneity; over broader size ranges, a mixed 1/*D* + 1/*D*^2^ form provides a more effective description. These results support a heuristic surface-driven interpretation of nanoscale structural disorder and clarify the different degrees of generality of the three scaling laws.

## Abstract 2

This work develops three surface-driven nanoscale models for the diffraction-derived lattice parameter, Debye–Waller coefficient and microstrain, clarifying why their size dependences are not equally universal. Atomistic simulations and literature data show that lattice parameter trends depend strongly on capillarity, chemistry and defects, whereas the Debye–Waller coefficient and microstrain follow more robust surface-disorder and surface-stress gradient scaling laws.

## 1. Introduction

Nanocrystalline materials exhibit size-dependent properties because their large surface-to-volume ratio makes the surface a dominant contributor to thermodynamic stability, atomic structure and physical response. As the characteristic dimension decreases, surface excess energy, reduced atomic coordination and finite-size effects increasingly perturb the equilibrium state of the crystal. This general framework is reflected in several classical examples: the melting temperature of metallic nanoparticles decreases markedly with decreasing size, the solubility of small particles increases according to capillarity-based thermodynamic arguments, and semiconductor quantum dots display size-tunable electronic and optical properties as a consequence of quantum confinement (Buffat & Borel, 1976 ▸; Kaptay, 2012 ▸; Brus, 1984 ▸). These well established phenomena show that nanocrystals cannot be regarded simply as smaller pieces of bulk matter, but rather as systems whose behaviour is increasingly governed by surface and interface contributions (Boles *et al.*, 2016 ▸).

Among diffraction-derived structural observables, the average lattice parameter is perhaps the most familiar example of a size-dependent quantity, but it is also one of the least universal. In metallic nanoparticles, classical capillary- and elasticity-based models predict lattice contraction with decreasing size, and this trend is well documented for systems such as Pd (Lamber *et al.*, 1995 ▸; Huang *et al.*, 2007 ▸). At the same time, extensive literature shows that the sign and magnitude of the effect are not general. Reviews of the field emphasize that lattice contraction is common in pure metals, whereas lattice expansion is frequently observed in ionic nanocrystals and metal oxides (Diehm *et al.*, 2012 ▸). Ceria is a particularly instructive case: lattice expansion with decreasing size has been related to Ce^3+^ formation and oxygen vacancies (Deshpande *et al.*, 2005 ▸), while more recent work has shown that adsorbed surface species can also dominate the observed size dependence (Prieur *et al.*, 2020 ▸; Subbareddy *et al.*, 2026 ▸). Anatase (TiO_2_) provides another clear example of non-universality, as the lattice parameters may evolve nonlinearly with size and even display opposite trends for the *a* and *c* axes (Swamy *et al.*, 2006 ▸; Ahmad & Bhattacharya, 2009 ▸). These examples indicate that the average lattice parameter reflects a competition between capillary stress, under-coordination-driven bond rearrangement, defect chemistry, and interactions between the nanocrystal surface and its environment, rather than a universal monotonic law.

By contrast, the size dependence of the Debye–Waller coefficient and of the microstrain appears considerably more systematic. The Debye–Waller coefficient, or equivalently the mean-square atomic displacement, generally increases as the nanocrystal size decreases because under-coordinated atoms in the outer region of the particle exhibit larger vibrational amplitudes and stronger static disorder than atoms in a bulk-like core. In this sense, the leading size effect is naturally controlled by the fraction of atoms belonging to a surface-perturbed layer. Likewise, the microstrain extracted from diffraction line broadening usually increases as the crystalline domain becomes smaller, reflecting the increasing importance of non-uniform lattice relaxation, local stress fields and structural disorder. Experimental and modelling studies on nanocrystalline systems such as Au, Ag, Al, TiO_2_, CeO_2_ and WO_3_ support this more systematic trend, even though the detailed exponent and prefactors depend on the specific material and analysis method (Sadaiyandi, 2009 ▸; Salim *et al.*, 2023 ▸; Deb & Chatterjee, 2020 ▸; Prieur *et al.*, 2020 ▸). This does not imply strict universality, but it suggests that the Debye–Waller coefficient and microstrain are structurally more robust probes of nanoscale disorder and surface-driven inhomogeneity than the average lattice parameter.

The present work is motivated by this contrast. Here, we use the non-universality of lattice parameter trends as a point of comparison but place emphasis on two quantities that appear to follow more general size-dependent behaviour: the isotropic Debye–Waller coefficient, described here through a core–shell model, and the microstrain, interpreted in terms of surface-stress inhomogeneity and its evolution with nanocrystal size. The goal is therefore to develop simple but physically transparent scaling models for spherical metallic nanocrystals of f.c.c. (face-centred cubic) Pd, b.c.c. (body-centred cubic) Fe and h.c.p. (hexagonal close-packed) Ti, and to assess their broader applicability using representative diffraction data from the literature. At the same time, more flexible fitting forms are introduced to account for deviations of real systems from the ideal leading-order limits.

## 2. Methodology

Spherical Pd, Fe and Ti nanocrystals with diameters ranging from approximately 4 to 50 nm were carved from their respective supercells using *VESTA* software (v4.6.0) (Momma & Izumi, 2008 ▸) and relaxed using molecular dynamics (MD) simulations. All simulations were performed with the *LAMMPS kokkos* package (Thompson *et al.*, 2022 ▸) on the HUNTER supercomputer adapted to APUs, using the metal unit system and standard embedded-atom-method (EAM) interatomic potentials (Zhou *et al.*, 2004 ▸; Mendelev *et al.*, 2003 ▸; Mendelev *et al.*, 2016 ▸). The nanoparticle structures were first energy-minimized by the conjugate-gradient method in order to remove residual stresses. They were then equilibrated at 300 K in the canonical (NVT) ensemble using a Nosé-Hoover thermostat with a time step of 0.005 ps. After equilibration, the simulations were continued in the microcanonical (NVE) ensemble, and atomic configurations were periodically saved for structural analysis. Before all post-processing steps, each particle was recentred at its centre of mass.

Two types of coordinate sets were used throughout the analysis. Instantaneous configurations contain both static structural disorder and thermal vibrations, whereas time-averaged coordinates were obtained by averaging atomic positions over a sufficiently long MD trajectory in order to suppress the thermal contribution and retain only the static distortion field. The comparison between these two data sets proved essential for separating static and dynamic contributions to diffraction.

Local stress was computed from the per-atom virial tensor. The hydro­static virial stress at each atom is defined as
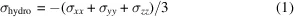
where σ_*xx*_, σ_*yy*_ and σ_*zz*_ are the diagonal components of the virial stress tensor. With this convention, positive values correspond to compressive hydro­static stress. Continuous stress fields were obtained by Gaussian-kernel interpolation of the per-atom virial values onto a regular grid, using a smoothing parameter α chosen to match the characteristic atomic-scale features for each particle size.

Powder diffraction patterns were calculated from the MD trajectories using the Debye scattering equation (DSE) as implemented in the *Debyer* code (Thomas, 2010 ▸) and were analysed with *TOPAS* (academic version v8.65) (Coelho, 2018 ▸). The refinements of the X-ray diffraction (XRD) patterns were done using a whole powder pattern modelling (WPPM) approach for line profile analysis (Scardi & Leoni, 2002 ▸; Scardi & Malagutti, 2024 ▸), and Sakuma thermal diffuse scattering models (Sakuma, 1995 ▸), similarly to what has been performed in our previous publications (Scardi & Gelisio, 2016 ▸; Scardi & Malagutti, 2024 ▸). Further technical details are provided in supplementary note 1 (see the supporting information).

## 3. MD results

### 3.1. Surface stress maps and internal strain fields

**Figure 1:**
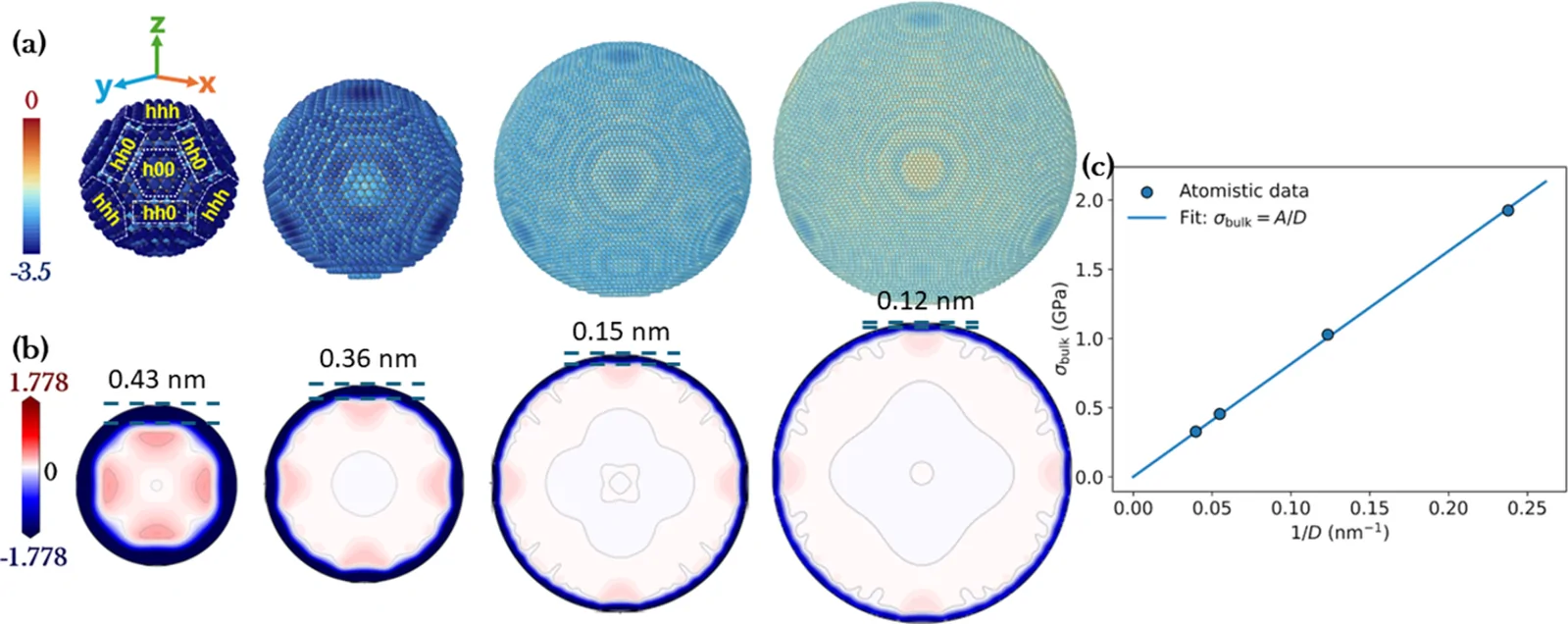
(*a*) Surface-projection stress maps for 4, 8, 18 and 25 nm Pd nanospheres and (*b*) their corresponding cross sections. Compressive stress = blue, tensile stress = red. The bars represent a rough estimation of the thickness of the compressive-stress surface shell. (*c*) Plot showing linear dependence of surface stress on inverse particle size.

Figs. 1 ▸(*a*) and 1 ▸(*b*) show the stress maps for nanocrystals with diameters from 4 to 25 nm. We observe that (i) the surface is in a general state of compression, due to the reduced coordination of the surface atoms; this corresponds to a state of average compression towards the interior of the nanocrystal that follows a 1/*D* law [Fig. 1 ▸(*c*)], in substantial agreement with surface thermodynamics and the Laplace equation. However, (ii) the stress state is not uniform: the faceting of the crystal, particularly evident at small dimensions, leads to surface stress gradients and to strain heterogeneity that extends into the particle interior. Therefore, there is an average compressive field, which tends to reduce the cell parameter as the dimensions decrease, but also a non-homogeneous surface stress field, which leads to variations in the strain within the crystal, *i.e.* to the presence of microstrain.

**Figure 2:**
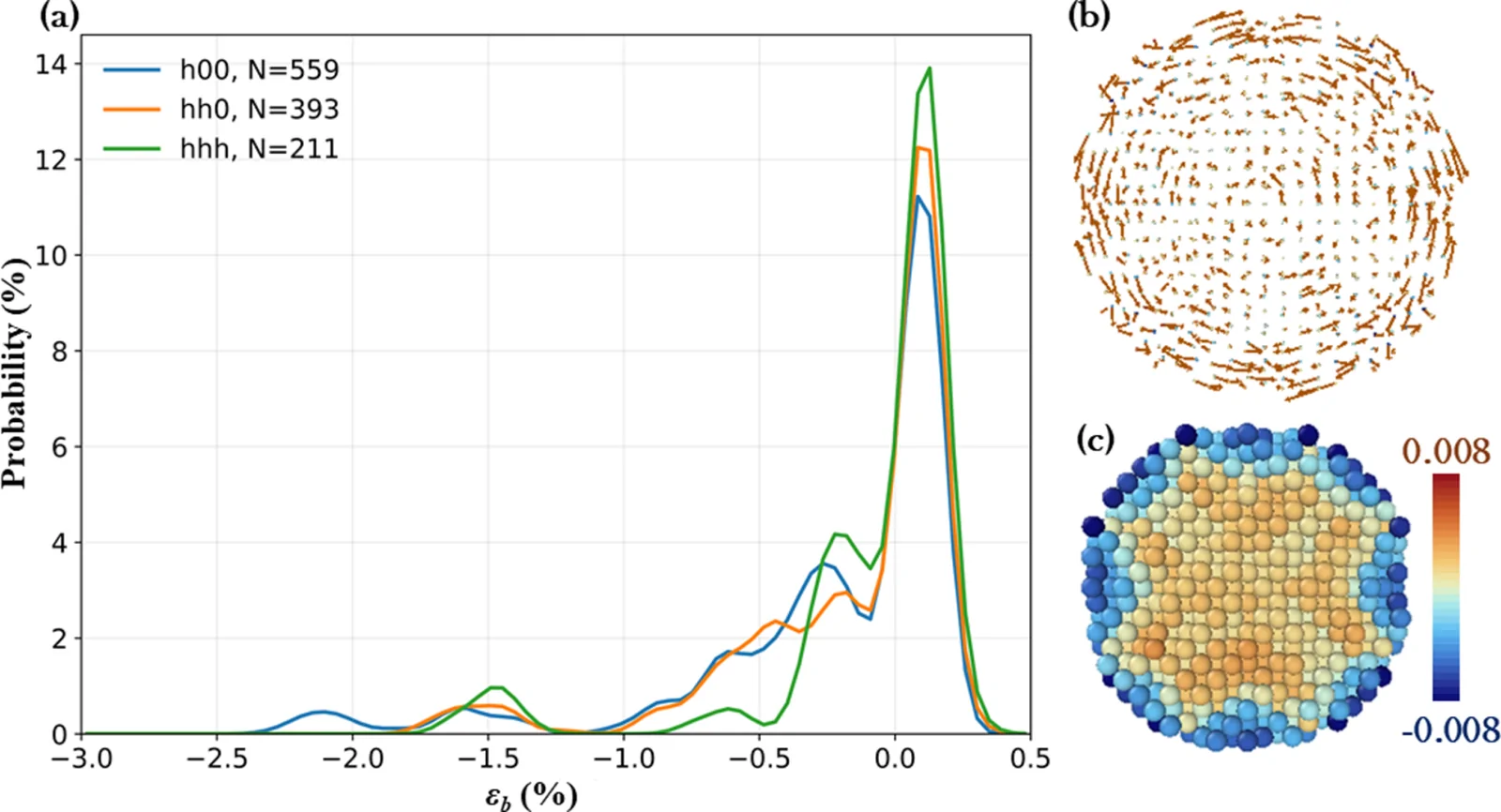
(*a*) Average local strain across the three cross sections 〈*h*00〉, 〈*hh*0〉 and 〈*hhh*〉; (*b*) tangential surface displacement field; (*c*) cross-sectional view of 4 nm Pd nanosphere showing the local strain variation from the core to the surface.

These features are even more evident in the strain distributions and maps shown in Fig. 2 ▸. The average local strain distributions for three families of crystallographic directions [〈*h*00〉, 〈*hh*0〉 and 〈*hhh*〉, Fig. 2 ▸(*a*)] in the 4 nm Pd nanosphere are broad and asymmetric [Fig. 2 ▸(*a*)]. The extended negative tails are noticeable, revealing the presence of strongly compressed local atomic environments. Despite the marked elastic anisotropy of palladium (Zener ratio *A_r_* = 2.8), the distributions are comparable in width, suggesting a microstrain that varies little with the crystallographic direction. Similar results are obtained for Fe and Ti (see supplementary note 2).

For the same Pd nanocrystal, the tangential displacement field in Fig. 2 ▸(*b*) shows that the surface relaxation is highly non-uniform. Instead of a uniform radial contraction that one might expect for an ideal spherical solid, the atomistic nature and surface faceting lead to localized displacement patterns across the nanoparticle surface. However, the displacement field is more spatially distributed because curvature partially averages the elastic anisotropy. At these sizes, most atoms are located within a few surface coordination shells, so local coordination differences strongly influence the displacement behaviour throughout the nanoparticle. This is particularly evident in Fig. 2 ▸(*c*), which shows the variation of local strain from the particle interior towards the surface. Larger strain fluctuations occur near the outer atomic layers, while the central region remains comparatively less distorted. Unlike larger nanoparticles, however, the strain perturbation is not confined to a thin surface shell. The strain heterogeneity extends through a substantial fraction of the entire nanoparticle volume, showing that the distinction between surface and bulk becomes increasingly unclear at ultrasmall sizes. This observation is consistent with the stress maps of Fig. 1 ▸, which show that surface-induced strain fluctuations penetrate deeply into the particle at small sizes.

### 3.2. Warren plots and the effective constant-microstrain approximation

The strain dispersion in the nanocrystal shown in Fig. 2 ▸ affects the diffraction profile. This can be described as a microstrain effect, *i.e.* the root mean square (RMS) strain 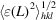. This is the width of the strain distributions for all pairs of atoms at a distance *L*, ranging from zero up to the maximum distance in the domain (the diameter *D* in this case), along each crystallographic direction [*hkl*] of interest for the powder pattern of the material. Equivalently, the effect can be described in terms of the RMS displacement for the pair distance *L*, 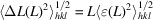, and corresponding pair displacement distributions. Up to constant factors and known terms (mainly Lorentz–polarization, Debye–Waller and structure factors), the diffraction peak for a nanocrystal powder can be modelled with good approximation as the Fourier transform of both size [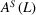] and microstrain contributions [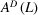],
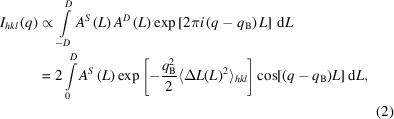
with 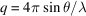 (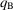 in the Bragg condition). For spherical crystalline domains
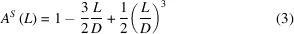
with 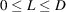.

**Figure 3:**
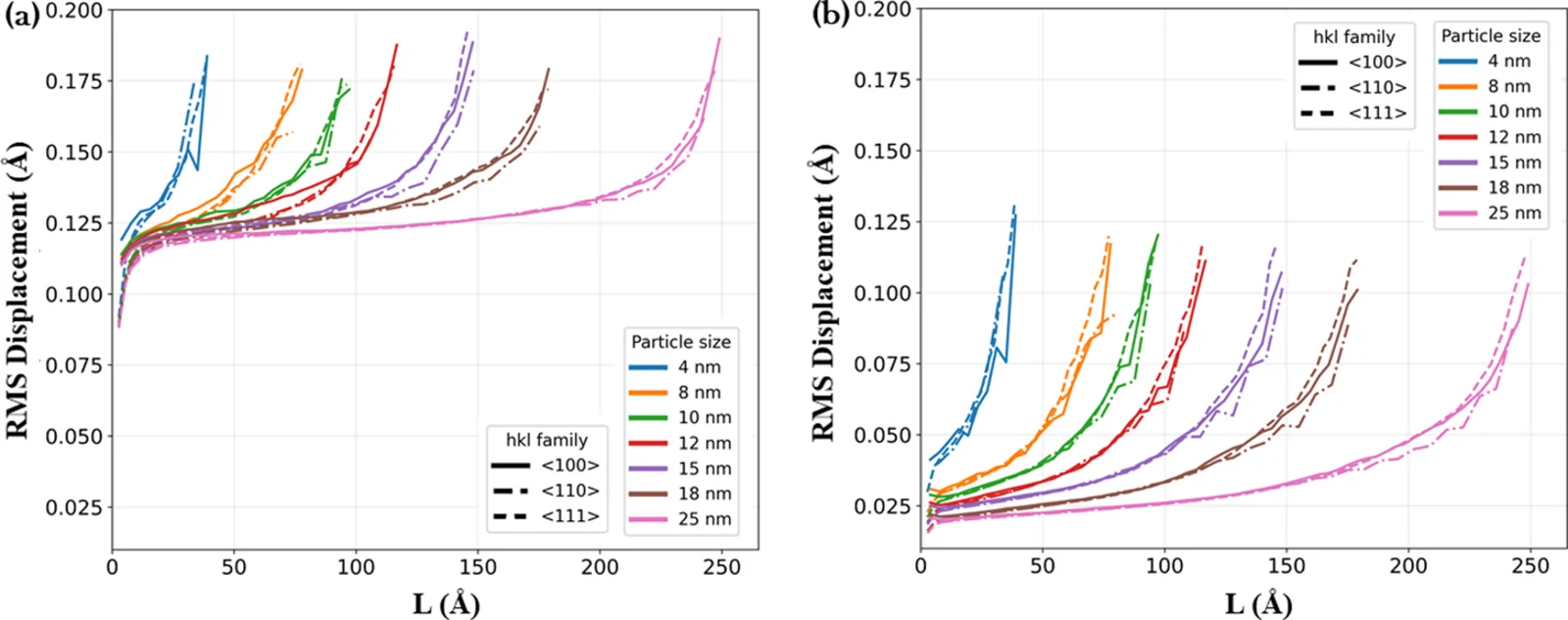
Warren plots of Pd nanospheres for (*a*) instantaneous coordinates, sampled for 100 frames of the steady-state MD simulation, and (*b*) time-averaged atomic coordinates. See text for details.

**Figure 4:**
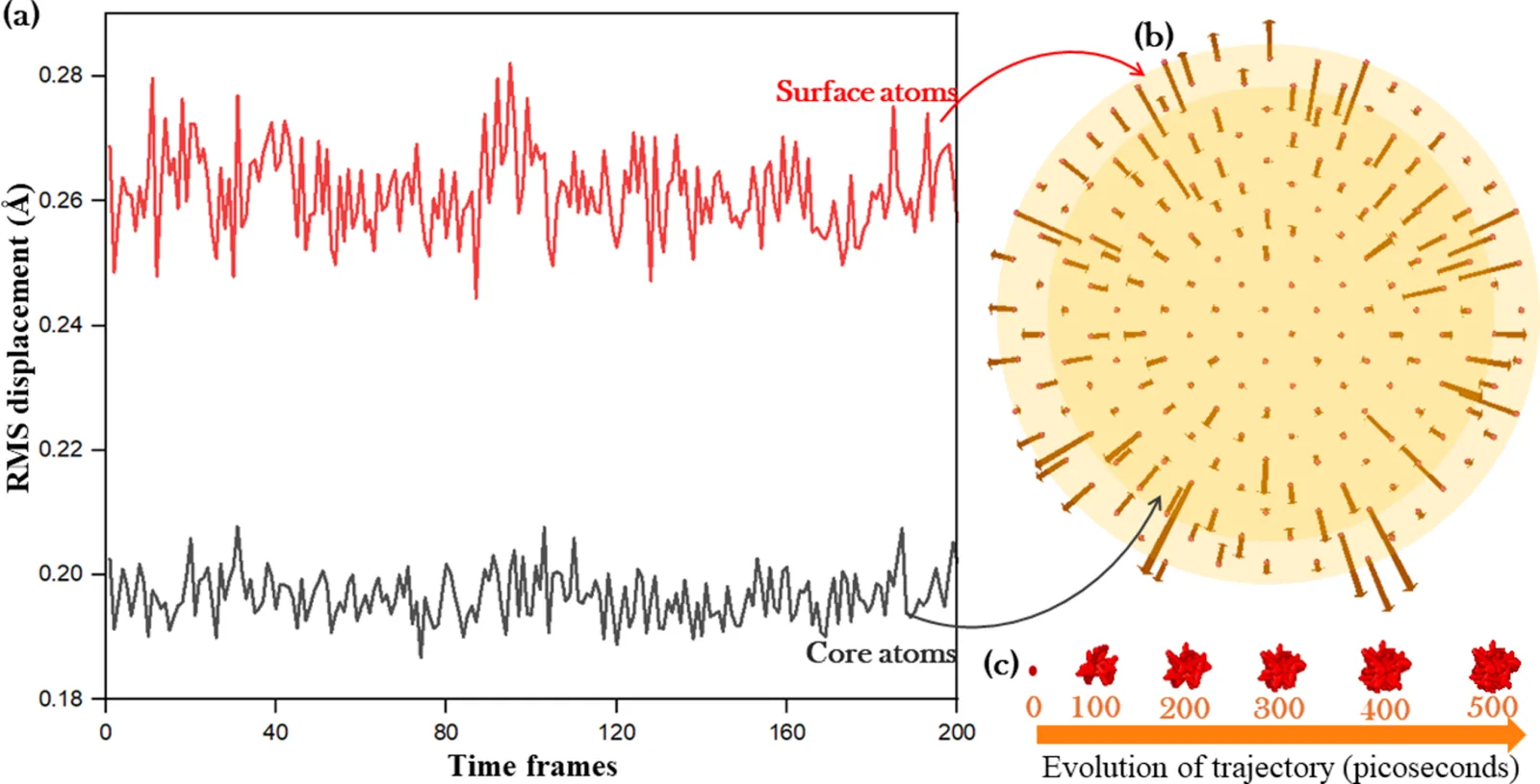
(*a*) Time-dependent RMS displacement of atoms at the core and the surface; (*b*) atomic displacement vectors on a cross section of the 4 nm Pd nanocrystal; (*c*) evolution of atomic trajectory.

MD simulations directly provide the RMS displacement as Warren plots (WP) (Warren & Averbach, 1950 ▸; Scardi *et al.*, 2025 ▸). This is shown in Fig. 3 ▸ for spherical Pd nanoparticles ranging from 4 to 25 nm in diameter. The plot in Fig. 3 ▸(*a*) is for instantaneous coordinates, sampled for 100 frames of the steady-state MD trajectory (*cf*. Fig. 4 ▸), whereas in Fig. 3 ▸(*b*) the WPs refer to time-averaged coordinates over the 100 frames of the MD trajectory. Thus Fig. 3 ▸(*a*) can be considered as a realistic model of WP from experimental measurement, including static and dynamic displacement effects; the latter are eliminated in Fig. 3 ▸(*b*), where the RMS displacement only considers static disorder. The difference between the two is approximately constant, a ‘step’, which represents the contribution of the dynamic disorder to the Debye–Waller (DW) coefficient, while the step for the static disorder [in Fig. 3 ▸(*b*)] is much smaller.

The RMS displacement increases with the distance *L* between atom pairs, with a slope, and hence also the corresponding microstrain, which increases for smaller nanocrystal sizes. In equation (2), the microstrain term 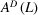 multiplies the size term 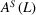, which is a function strongly decreasing with *L* [from equation (3), 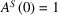 and 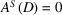]. This implies that for the purposes of the diffraction profile the WP trend weighs more for the shorter distances *L*, which show an approximately linear trend and therefore an approximately constant microstrain. This approximation is widely used in the most popular algorithms and software for line profile analysis and the Rietveld method, *e.g.* in the double-Voigt approach of *TOPAS* (Coelho, 2018 ▸). In the supplementary note 3, in addition to the WP for Fe and Ti, the comparison between modelling of the diffraction patterns with the trends in Fig. 3 ▸ and corresponding linear approximations for Pd is described. Since the main effect on the line profile is that of the size, due to the small dimensions of the crystalline domain, the linear approximation is reasonable for discussing microstrain effects in the context of the models described in the next sections.

### 3.3. Surface-enhanced atomic displacements and the DW contribution

MD simulations offer another important detail for nanocrystal diffraction, related to the amplitude of atomic vibrations. The atomic RMS displacement is shown in Fig. 4 ▸(*a*) along the MD trajectory over 200 frames. The system progressively reaches a steady state during the simulation, and the last 100 frames are used for the simulation of the powder diffraction pattern. Fig. 4 ▸(*b*) presents a cross-sectional displacement vector map of the same 4 nm Pd nanoparticle. The RMS displacement is significantly higher in the surface region, resulting in an average increase in the DW coefficient for the nanoparticle, directly related as 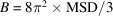, with MSD being the mean square displacement. Fig. 4 ▸(*c*) illustrates the trajectory of a representative surface atom extracted at different stages of the MD simulation, demonstrating the evolution of its vibrational motion with time. For a sufficiently long period of time, the shape of its trajectory in space is revealed to be spherical, meaning that the average atomic movement is isotropic.

## 4. Nanoscale models

The models introduced here describe how the diffraction-derived structural parameters evolve with nanocrystal size: the average lattice parameter, the isotropic DW coefficient and the microstrain. As shown in the previous paragraph, even if all three are influenced by the growing importance of the surface at small size, they do not arise from the same microscopic mechanism. The lattice parameter is mainly controlled by the average capillary contribution stress associated with the particle surface free energy, the DW coefficient reflects the increasing statistical weight of surface atoms with enhanced vibrational and static disorder, and the microstrain originates from spatially non-uniform strain fields generated by surface-stress inhomogeneity. The aim is not to formulate universal laws, but rather to establish simple leading-order scaling models for nearly spherical nanocrystals, to be compared later with atomistic simulations and literature data. More detailed derivations are given in supplementary note 4.

### 4.1. Lattice parameter

For an ideal spherical nanoparticle of diameter *D*, surface thermodynamics implies a Laplace-like internal pressure that scales inversely with size. In a continuum description, this pressure may be written in terms of an orientation-averaged surface free energy *g* as
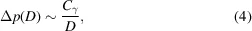
where 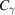 is a geometric coefficient proportional to *g* and dependent on particle shape. For an elastically isotropic sphere with bulk modulus *K*, this pressure variation [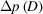] produces a volumetric strain
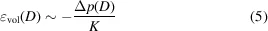
corresponding to a linear isotropic strain
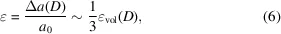
where 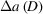 corresponds to the variation of the lattice parameter caused by the microstrain and 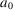 the original lattice parameter. The average lattice parameter therefore follows the leading-order form
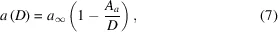
with 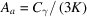, and 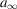 corresponding to the bulk value of the lattice parameter. Equation (7) is the correct leading-order result for the idealized case of clean, isolated, approximately spherical nanocrystals whose average strain is dominated by the capillary surface contribution, in agreement with the results of Fig. 1 ▸. The same 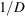 dependence emerges from the elasticity treatment of Huang *et al.* (2007 ▸), who showed that the effect becomes small for sufficiently large Pd particles. However, this expression should not be regarded as a universal law as it depends on the grain boundary and surface mechanisms specific for each system.

### 4.2. Debye–Waller coefficient

The DW coefficient is treated with a simple core–shell model, a reasonable approximation of what is shown in the previous paragraph and in Fig. 4 ▸ in particular. Let the nanocrystal have an outer surface-perturbed shell of thickness *t*, typically of the order of a few Å. The fraction of atoms belonging to this shell is
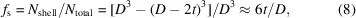
with *t* << *D*. Thus, the fraction of atoms affected by surface disorder scales as 1/*D*. Let 〈*u*^2^〉_b_ and 〈*u*^2^〉_s_ be the mean-square displacements of bulk-like core atoms and surface-shell atoms, respectively. Since surface atoms are under-coordinated and experience both enhanced vibrational amplitudes and stronger static relaxation disorder, one expects 〈*u*^2^〉_s_ > 〈*u*^2^〉_b_ [see again Fig. 4 ▸(*a*)]. The particle-averaged MSD is
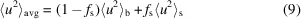
which after substitution of equation (8) becomes
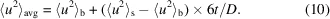
As a consequence, the isotropic DW coefficient is described as
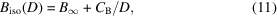
where *B*_∞_ is the bulk DW coefficient and *C*_B_ = 16π^2^*t* × (〈*u*^2^〉_s_ − 〈*u*^2^〉_b_) is a positive constant. Compared with the lattice parameter model, equation (11) is more robust because it does not depend on the sign of an average strain: it only requires that the outer shell be more disordered than the bulk-like core. This is why the increase of the DW coefficient with decreasing nanocrystal size is expected to be more general than the corresponding trend in the lattice parameter.

### 4.3. Microstrain from surface-stress inhomogeneity

Microstrain in diffraction line profile analysis represents a distribution of local lattice spacings rather than a mean shift. As already pointed out in the previous paragraph, the effective scalar microstrain ɛ_micro_ may be viewed as the RMS amplitude of the inhomogeneous part of the strain field within a coherently diffracting domain. This inhomogeneous component is associated with the non-uniform surface stress field generated by faceting, edges, vertices and local variations in atomic coordination, particularly relevant at the nanoscale.

Let τ_surf_(*s*,*D*) denote the local surface stress along the nanocrystal surface, where *s* is a coordinate along the boundary. We define Δτ_surf_ as the typical variation in surface stress between distinct surface regions, clearly shown in Figs. 1 ▸ and 2 ▸, and σ_0_(*D*) as the characteristic internal stress scale induced by the surface boundary conditions. At the level of scaling laws, it is reasonable to assume that
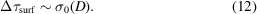
For small, strongly faceted nanocrystals, the characteristic lateral distance over which the surface stress changes is of order *D*. The corresponding gradient along the surface then scales as
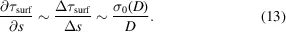


Since the characteristic surface-induced internal stress scale behaves as
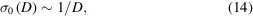
we obtain
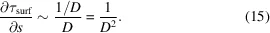
Thus, surface-stress heterogeneity generates a surface-stress gradient of order 1/*D*^2^. If the corresponding local strain fluctuations extend over a substantial fraction of the nanocrystal volume, as shown in Figs. 1 ▸ and 2 ▸, the RMS microstrain follows the same leading dependence:
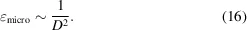
This is the natural small-particle limit for strongly faceted nanocrystals, where surface-stress variations are controlled by the global particle size. A uniform hydro­static strain would shift the diffraction peaks but would not broaden them; therefore, the observed microstrain reflects the inhomogeneous part of the strain field rather than its mean value.

As suggested by Fig. 1 ▸, for larger particles surface-stress inhomogeneities become more localized, and the associated strain field may no longer occupy a uniformly weighted fraction of the volume, while contributions from bulk defects, finite strain correlation lengths and particle shape become relatively more important. In that case, the effective decay of the measured microstrain can become slower than the ideal 1/*D*^2^ limit over the accessible size range. For practical fitting of simulated or experimental data, we therefore adopt the mixed form
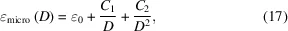
where ɛ_0_ is a residual size-independent baseline (instrumental or bulk defect contribution), *C*_1_/*D* represents a weaker large-*D* correction and *C*_2_/*D*^2^ is the leading small-particle term associated with surface-stress gradients. Equation (17) is used here as a physically motivated empirical expression for the crossover from strongly surface-dominated behaviour at small size to weaker and more localized size effects at larger size.

## 5. Results and discussion

### 5.1. Nanoscale models applied to simulated Pd, Fe and Ti nanocrystals

The nanoscale models of the previous section were tested on the results of MD simulations. The instantaneous coordinates were used to generate the powder patterns with the DSE, subsequently averaged to obtain a good pr­oxy of an experimental measurement. The patterns were then analysed with *TOPAS*, using a standard mode, both for the structural information of the three metals and for the microstructural information. For the latter, the specific macro for the size broadening effect from spherical domains was used [based on equation (3)], while for the microstrain the standard command (e0_from_Strain) was used, which assumes a constant microstrain (Dinnebier *et al.*, 2018 ▸). Specifically, the *TOPAS* analysis demonstrated that even if both Lorentzian and Gaussian components were included for the microstrain effect on the profile, the Gaussian component was sufficient to model these data (and similarly for Fe and Ti), thus requiring a single parameter. As predicted by the MD results in Section 3, it is not even necessary to introduce elastic anisotropy parameters for these spherical nanocrystals.

**Figure 5:**
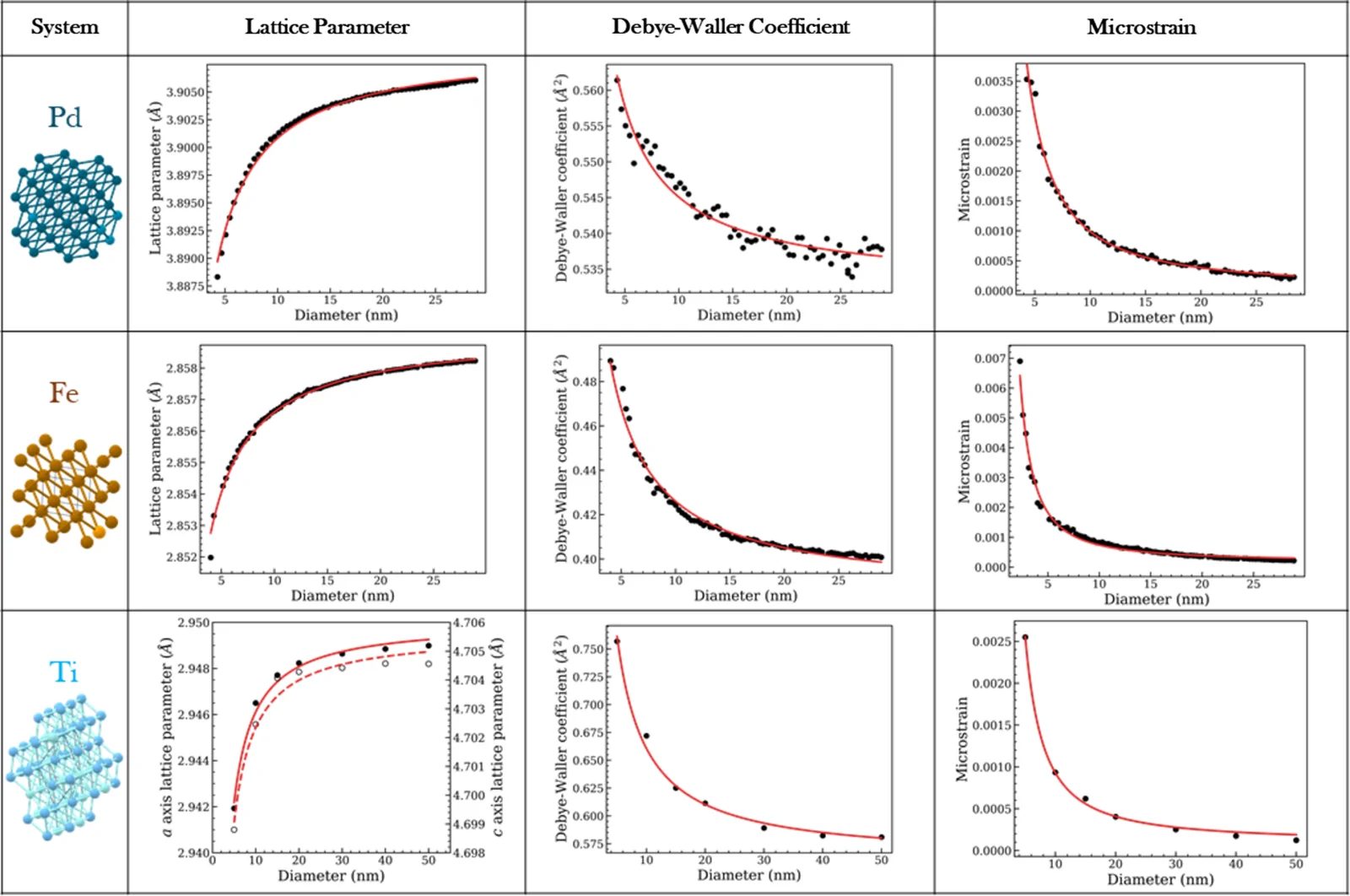
Variation of structural parameters – lattice parameter, DW coefficient and microstrain – with crystallite size in simulated nanocrystals of Pd, Fe and Ti. Data from XRD powder patterns simulated with DSE using MD results (dots) and nanoscale models described in Section 4 (line). For Ti, dashed line for *c* axis, full line for *a* axis of the h.c.p. unit cell.

The results are summarized in Fig. 5 ▸, for lattice parameter, DW and microstrain coefficients, together with the trends of equations (7), (11) and (17), respectively, refining the free parameters using least squares. Numerical details are reported in supplementary note 5. Nanoscale models perform well, confirming the plausibility of their underlying hypotheses and their sufficient flexibility. A more robust assessment is presented in the next section, based on experimental data.

### 5.2. Nanoscale models applied to experimental results

Literature studies including several experimental investigations on nanocrystalline materials have shown that structural parameters such as the lattice parameter, DW coefficient and microstrain evolve systematically with decreasing particle size, although the trends are not equally universal. Reported lattice parameter behaviour varies strongly between materials and synthesis conditions, reflecting the combined influence of surface stress, defects, oxidation and anisotropic relaxation. In contrast, increases in the DW coefficient and microstrain are observed much more consistently across both metallic and oxide nanoparticles, indicating a general enhancement of structural disorder at small sizes. To evaluate the broader applicability of the proposed scaling relations, the nanoscale models are compared below with representative experimental literature data spanning f.c.c. metals, complex oxides and a magnetic intermetallic system. Numerical details of the data fits are reported in supplementary note 5.

**Figure 6:**
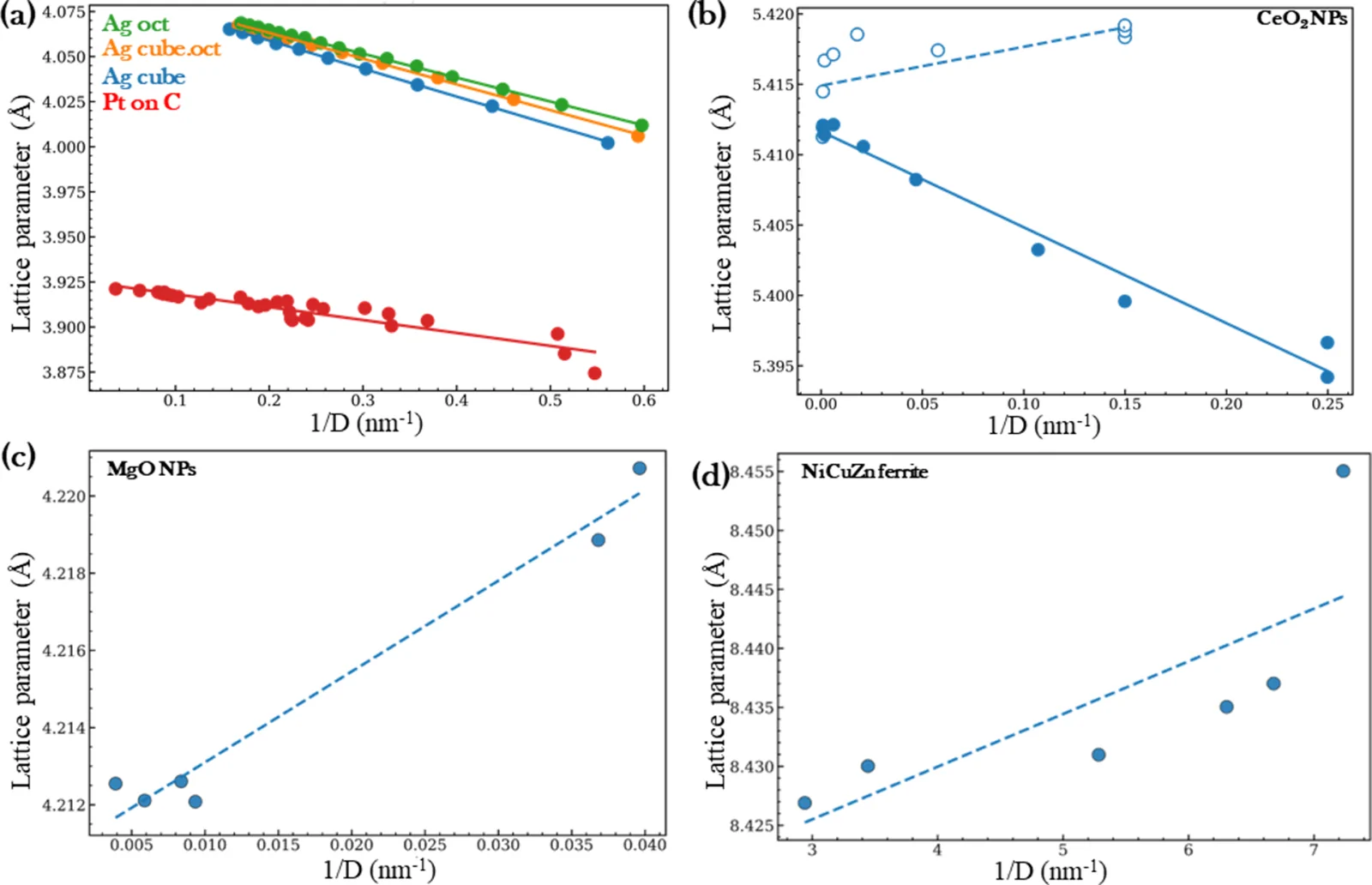
(*a*) Lattice parameter variation with increasing particle size according to the 1/*D* law for metal systems: Ag cubes, octahedron and cuboctahedron (Chen *et al.*, 2017 ▸); Pt on carbon support (Leontyev *et al.*, 2014 ▸); (*b*) CeO_2_ lattice contraction (open circles, dashed lines) and expansion (filled circles, solid line) with increasing crystallite size (Chen *et al.*, 2010 ▸); (*c*) MgO nanoparticles (Barad *et al.*, 2024 ▸); (*d*) NiCuZn ferrite (He *et al.*, 2015 ▸); dashed lines are guides to the eye. All the data were digitized from the above-mentioned references.

The lattice parameter case studies [Fig. 6 ▸(*a*)] show that metallic nanoparticles generally approach the bulk lattice constant as particle size increases, but the magnitude of contraction at small sizes depends strongly on particle morphology and surface structure. In Ag nanoparticles, cubic particles exhibit the largest lattice contraction, while octahedral particles remain closest to the bulk value over the entire size range. The study (Chen *et al.*, 2017 ▸) directly correlates this behaviour with surface energy, showing that particles with higher surface energy undergo stronger structural contraction. Cubic Ag nanoparticles, dominated by 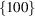 facets, therefore contrast more strongly than octahedral particles with predominantly 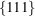 surfaces. Similar inverse-size behaviour is reported for Pt nanoparticles, where the lattice parameter decreases significantly below 10 nm and deviates by about 0.7% from the bulk value near 2 nm. However, the Pt literature also highlights substantial experimental variability, including reports of lattice expansion and weak size dependence, demonstrating that lattice evolution is highly sensitive to support interactions, defects and synthesis conditions rather than particle size alone (Leontyev *et al.*, 2014 ▸).

A more complex behaviour is observed in systems where surface effects become comparable with or dominate the bulk compression shown by Fig. 1 ▸ and predicted by equation (7). In CeO_2_, both lattice expansion and contraction have been reported, depending on synthesis conditions and surface chemistry [Fig. 6 ▸(*b*)] (Chen *et al.*, 2010 ▸). The cited study shows that oxygen vacancies, hydroxyl groups and surface relaxation collectively influence the lattice parameter, producing a non-monotonic size dependence at small crystallite sizes. As shown in Fig. 6 ▸(*b*), defect-related lattice expansion becomes increasingly important in ultrasmall particles because the fraction of surface and near-surface atoms increases strongly with decreasing size (Chen *et al.*, 2010 ▸). Similar non-monotonic behaviour is reported for MgO nanocrystals (Barad *et al.*, 2024 ▸), shown in Fig. 6 ▸(*c*), where an initial lattice contraction is followed by expansion in the low nanometre regime. The authors attribute this crossover to the competition between surface-induced contraction and weakening of ionic bonding in very small crystallites. In contrast, NiCuZn ferrites [Fig. 6 ▸(*d*)] (He *et al.*, 2015 ▸) mainly show lattice changes associated with sintering-induced densification, grain growth and cation redistribution rather than a direct nanoscale size effect.

These case studies demonstrate that lattice evolution in nanocrystals is highly material dependent and can be strongly influenced by surface chemistry, defect concentration, bonding character and synthesis conditions (He *et al.*, 2015 ▸; Chen *et al.*, 2010 ▸; Boles *et al.*, 2016 ▸). The nanoscale model of equation (7) is valid only if the assumptions of the Laplace equation [equation (4)] hold, and if the surface free energy plays an exclusive or at least relevant role. If other effects are present, the behaviour of the lattice parameter may be opposite, and not necessarily linear with 1/*D*, as in the last three cases considered.

**Figure 7:**
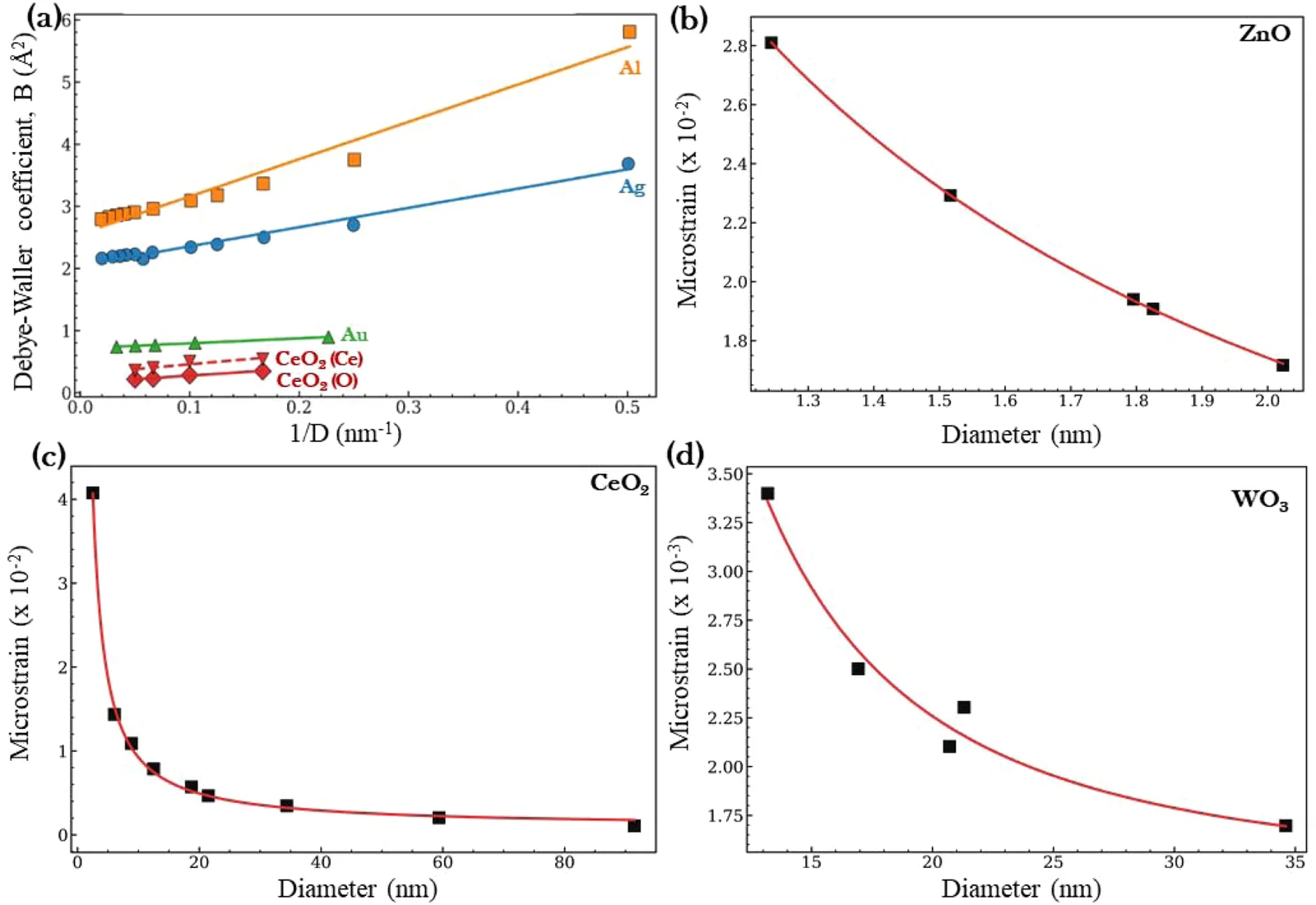
(*a*) Variation of DW coefficients with size for Al (Sadaiyandi, 2009 ▸), Ag (Sadaiyandi, 2009 ▸), Au (Batyrow *et al.*, 2023 ▸) and CeO_2_ (Subbareddy *et al.*, 2026 ▸) nanoparticles. Microstrain as a function of nanoparticle diameter for (*b*) ZnO (Prabakar *et al.*, 2021 ▸), (*c*) CeO_2_ (Prieur *et al.*, 2020 ▸) and (*d*) WO_3_ (Salim *et al.*, 2023 ▸). See text for details. All the data were digitized from the above-mentioned references.

The DW coefficient shows a much more consistent size dependence than the lattice parameter. In all reported cases shown in Fig. 7 ▸(*a*), *B* increases with 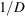. This trend is clear for Ag, Al and Au, where the approximately linear increase of *B* with 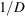 indicates that the surface contribution scales with the surface-to-volume ratio, in agreement with the MD results of Fig. 4 ▸ and the core–shell model of equation (11).

Reduced surface coordination weakens the local restoring forces and allows larger vibrational amplitudes (Sadaiyandi, 2009 ▸). This interpretation is also supported by MD simulations on Au nanocrystals (Batyrow *et al.*, 2023 ▸), where the MSD was found to be higher than the bulk value for all particle sizes and to decrease progressively as the diameter increased from 5 to 30 nm. The same study also showed that MSD increases from the particle centre towards the surface, confirming that the enhanced DW coefficient is mainly a surface-shell effect rather than a uniform bulk-like change. The CeO_2_ data follow the same qualitative behaviour, but the cation and anion sublattices contribute differently (Subbareddy *et al.*, 2026 ▸). The oxygen DW coefficient is larger and more size-sensitive than that of cerium, which is expected because oxygen atoms in oxide surfaces are weakly constrained and more affected by vacancies, hy­droxy­lation and local coordination disorder than cerium cations. Unlike the lattice parameter, the DW coefficient is a more direct measure of nanoscale disorder because smaller crystallites contain a larger fraction of dynamically disordered surface atoms, leading to higher *B* values regardless of average lattice expansion or contraction.

The microstrain data sets of Figs. 7 ▸(*b*) (Prabakar *et al.*, 2021 ▸), 7 ▸(*c*) (Prieur *et al.*, 2020 ▸) and 7 ▸(*d*) (Salim *et al.*, 2023 ▸) show a strong and systematic increase with decreasing crystallite size. The trends are different, but equation (17) provides a satisfactory effective description in all three cases. Additional contributions may of course arise from environmental interactions, defect accumulation, contamination and other thermodynamically favoured sources of lattice disorder at small size.

### 5.3. Extension to non-spherical particles: Pd nanocubes

**Figure 8:**
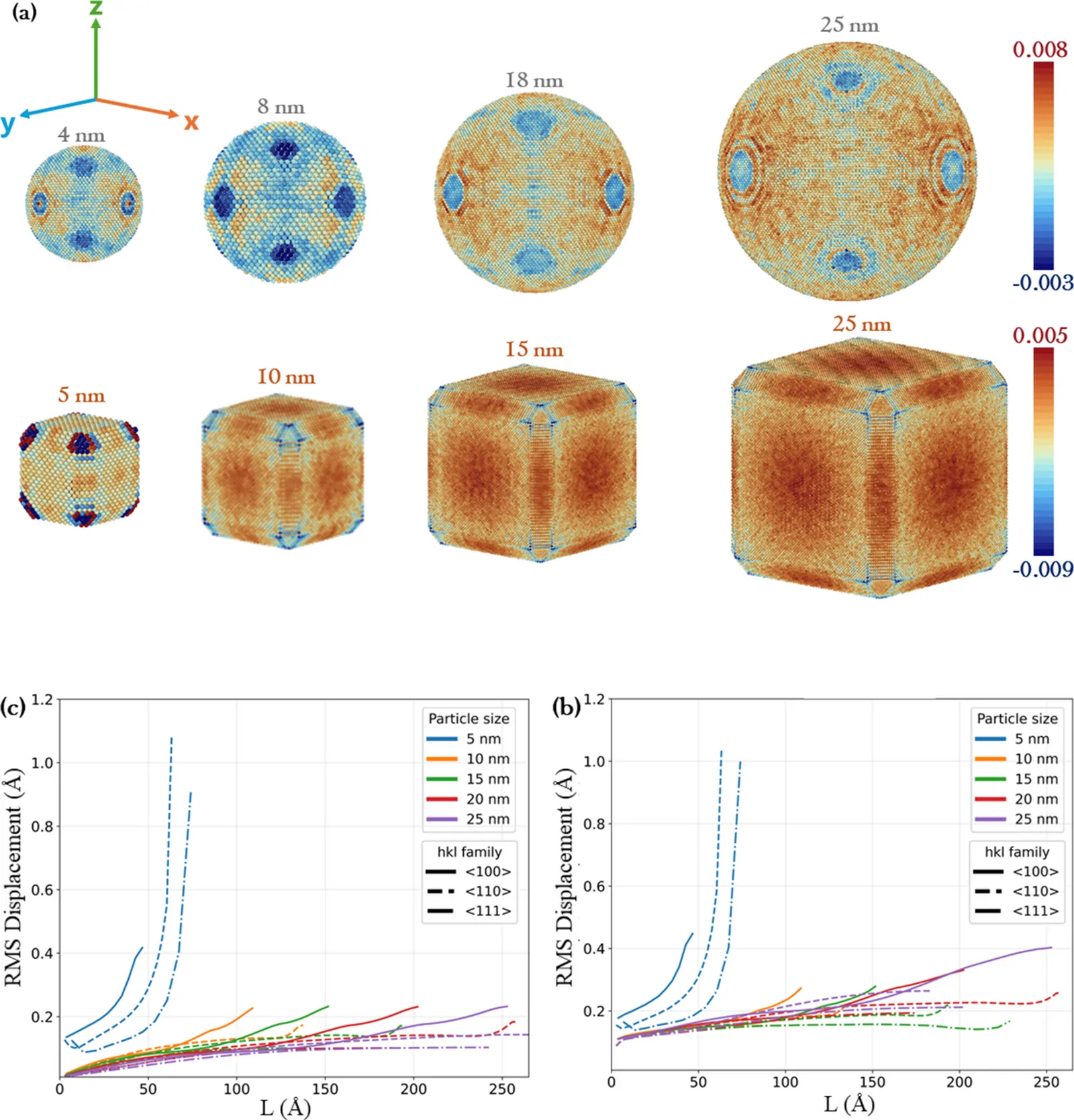
(*a*) Surface strain maps of Pd nanospheres (upper row) and Pd nanocubes (middle row) with increasing particle size. Colours represent the local strain distribution relative to the equilibrium structure. WPs for the nanocubes (bottom line) of different size, along the same three crystallographic directions, 〈100〉, 〈110〉, 〈111〉. The WPs are obtained as for the nanospheres of Fig. 3 ▸, from (*b*) instantaneous coordinates, sampled for 100 frames of the steady-state MD simulation, and (*c*) time-averaged atomic coordinates.

Inhomogeneous surface stress is always present, even for shapes very different from the spherical one. Fig. 8 ▸(*a*) illustrates this by comparing surface strain maps of Pd nanocrystals of spherical and cubic shapes (with truncated corners and edges), and the corresponding Warren plots [Figs. 8 ▸(*b*), 8 ▸(*c*)]. The RMS displacement trend is far from constant, implying relevant microstrain. The slopes increase with decreasing size in a qualitatively analogous way to that observed in Fig. 3 ▸ for spherical domains, even if the non-spherical shape carries significant anisotropy, as also observed experimentally by Scardi *et al.* (2015 ▸). This contribution to the presence of microstrain therefore appears to be governed by a law of rather general validity. Nanospheres show nearly isotropic displacement correlations, whereas nanocubes retain strong crystallographic anisotropy, but in all cases a microstrain appears as a consequence of the surface-stress inhomogeneity, increasing for smaller sizes.

## 6. Related literature

The following references are cited in the supporting information: Cullity (1978 ▸), Guinier & Fournet (1955 ▸), Saini & Khatri (2025 ▸), Scardi *et al.* (2004 ▸), Scardi *et al.* (2018 ▸), Tsunekawa *et al.* (2000 ▸), Wilson (1949 ▸).

## 7. Conclusion

Based on atomistic simulations and representative literature data, three simple nanoscale hypotheses have been formulated to describe the size dependence of the lattice parameter, Debye–Waller coefficient and microstrain in nanocrystals.

The lattice parameter follows the expected 1/*D* trend under ideal capillarity-driven conditions, as confirmed by MD results for spherical Pd, Fe and Ti nanocrystals. Comparison with experimental data, however, confirms that this behaviour is not universal, and may be weakened, reversed or rendered non-monotonic by surface chemistry, defects, non-stoichiometry and particle morphology. By contrast, the Debye–Waller coefficient exhibits a more systematic 1/*D* increase with decreasing size, consistent with the growing contribution of a surface shell of under-coordinated atoms characterized by enhanced vibrational amplitudes and static disorder. Microstrain likewise increases as size decreases, but its origin lies in surface-stress heterogeneity: in the small-particle limit, where strain fluctuations extend through a substantial fraction of the particle volume, the natural leading behaviour is 1/*D*^2^, whereas over broader size ranges a mixed 1/*D* + 1/*D*^2^ form provides a more effective description.

More generally, the results indicate that surface-stress gradients are an intrinsic consequence of nanoscale shape, faceting and reduced coordination, and therefore constitute a natural source of microstrain even in nominally defect-free particles. Although the present models were developed for spherical nanocrystals, the same physical mechanisms are expected to remain relevant in non-spherical systems, where anisotropy and strain localization become more pronounced.

## Supplementary Material

Supporting information. DOI: 10.1107/S2053273326007345/ae5184sup1.pdf

## Data Availability

All data analysed during this study are included in this published article (and the supporting information). The raw data sets and related files are available from the corresponding author upon request.
