# Supplementary material for: Surface-driven size scaling of the lattice parameter, Debye–Waller coefficient and microstrain in nanocrystals
Source: Acta Crystallogr A Found Adv. 2026 Aug 3;82(Pt 5):366–76. doi: 10.1107/S2053273326007345 (PMC13542917; doi:10.1107/S2053273326007345)
Supplement: Supplementary file 1 [file a-82-00366-sup1.pdf]

FOUNDATIONS
ADVANCES

Volume 82 (2026)

Supporting information for article:

Surface-driven size scaling of the lattice parameter, Debye–Waller coefficient and microstrain in nanocrystals

Sirisha Subbareddy, Erick Agnolin, Marcelo Augusto Malagutti, Narges Ataollahi, Robert Dinnebier and Paolo Scardi

Supplementary Note 1: Powder diffraction patterns

Molecular dynamics simulations were performed for three different systems: Palladium (Pd), Iron (Fe) and Titanium (Ti), using the EAM potentials and NVT followed by NVE ensembles provided the coordinates of all the simulated atoms for 100 snapshots during the NVE calculations. With the coordinates, a histogram distribution of pair distances was calculated which served as an input to calculate the Debye scattering equation using Debyer. Two types of average were performed: i) static average obtained by averaging the atomic coordinates for all the simulated frames and ii) the time average, obtained by averaging the total scattering patterns of each snapshot. More details are provided in Ref (Scardi & Malagutti, 2024).

The calculated diffraction intensity is refined using line profile formalism incorporating finite size broadening using the WPPM common volume function for a sphere (Scardi *et al.*, 2018). Small angle scattering contributions were modelled using spherical shape expressions (Guinier *et al.*, 1955). The microstrain broadening was fitted using Gaussian line profile, relating the peaks integral breadth to a tangent 2θ Stokes & Wilson rule for microstrain (Cullity, 1978; Scardi *et al.*, 2004; Wilson, 1949). TDS was modelled using the Sakuma approach (Sakuma, 1995) refining the correlation coefficients for the nearest sixteen coordination shells. The TDS used the same Debye-Waller coefficients as the one modelled in the Bragg part. The structural and microstructural parameters – radius of the sphere, lattice parameter, Debye-Waller coefficient and the microstrain are refined during fitting. The refinement quality of the fit was evaluated using the weighted-profile residual (R_{wp}) and goodness-of-fit (gof) parameters reported by TOPAS. A sample fit of the powder diffraction pattern for a 10 nm Pd nanosphere (Figure S1) is shown below:

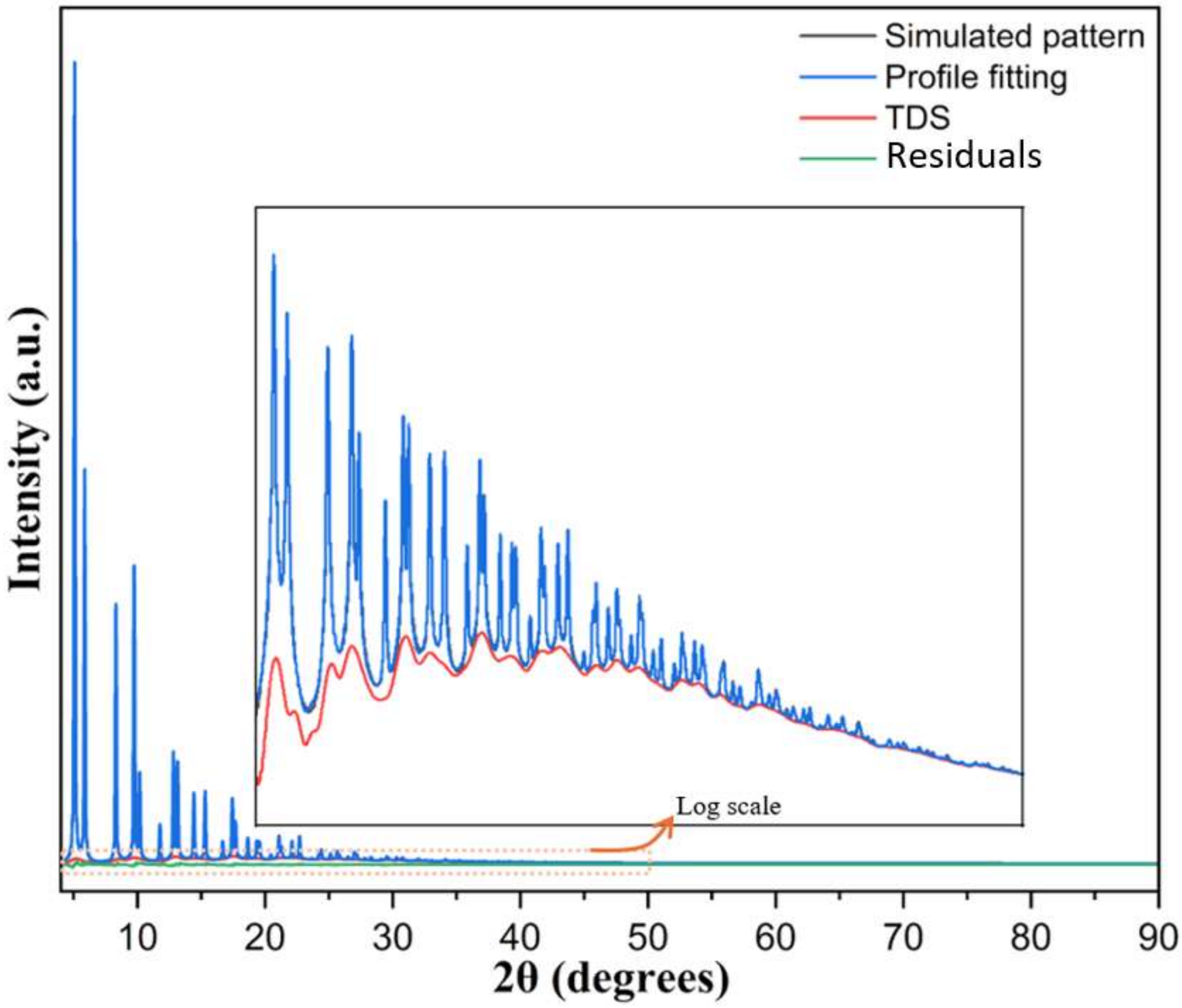

Figure S1: WPPM fitting of the X-ray diffraction profile of a 10 nm Pd nanosphere using TOPAS software. Inset represents the diffraction intensity on a logarithmic scale over the 2θ range 4° - 50°. (R_{wp} = 2.65 %)

Supplementary Note 2: Surface stress maps

The surface stress maps, their corresponding cross-sectional maps and the local strain distribution for the three crystallographic directions for the Fe and Ti systems are illustrated in Figures S2 and S3, respectively. Items a in both figures show the overall surface stress on the nanospheres of Fe and Ti systems respectively. Non-homogeneity and domination of the compressive stress on the surface of the nanospheres is evident from these Figures. The cross-sectional images of these systems are represented in their respective items b. Compared to the case of palladium discussed in Section 3.1 of the manuscript, Ti exhibits more directional dependence and stronger facet-to-facet contrast. These features are more evident in the local strain distributions shown in Figures S2c and S3c. All the procedures followed here to obtain the Figures are described in References (Tsunekawa *et al.*, 2000; Scardi *et al.*, 2015).

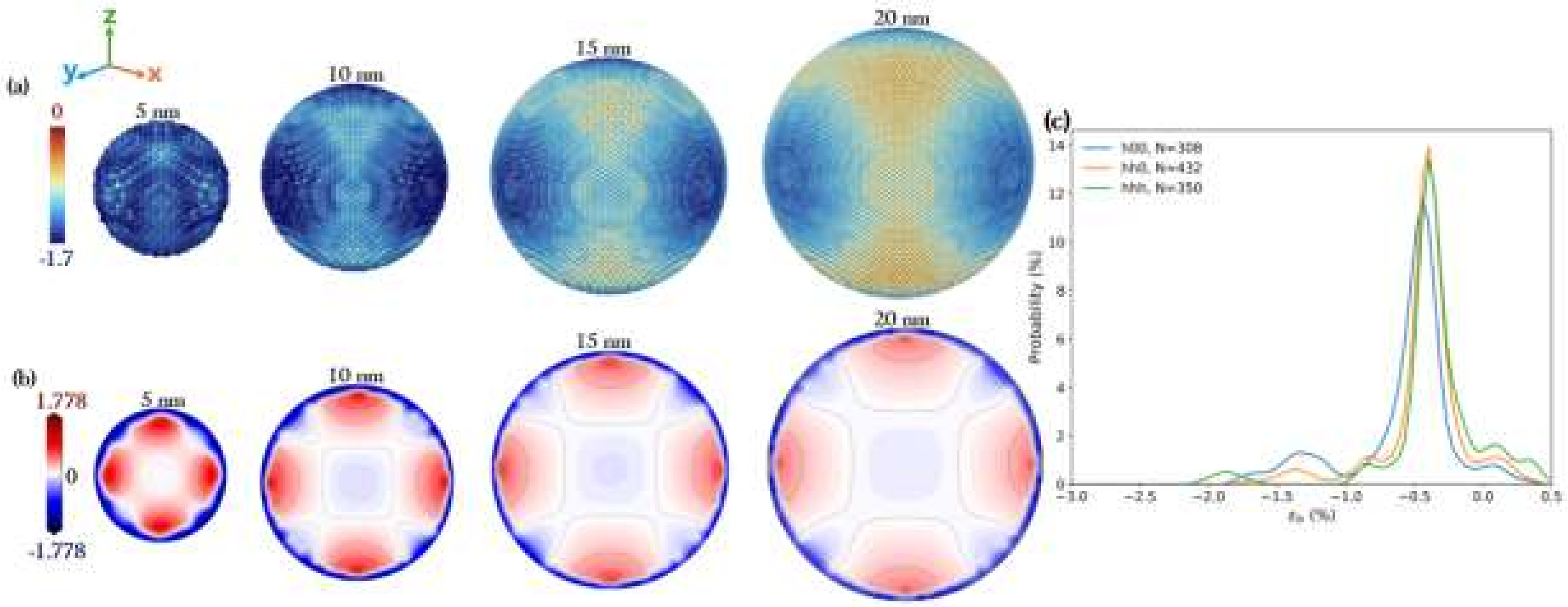

Figure S2: (a) *Surface-projection stress maps for 5, 10, 15 and 20 nm Fe nanospheres and (b) their corresponding cross sections. Compressive stress = blue, tensile stress = red. (c) Average local strain across the three cross sections (hh0) (hh0) and (hhh).*

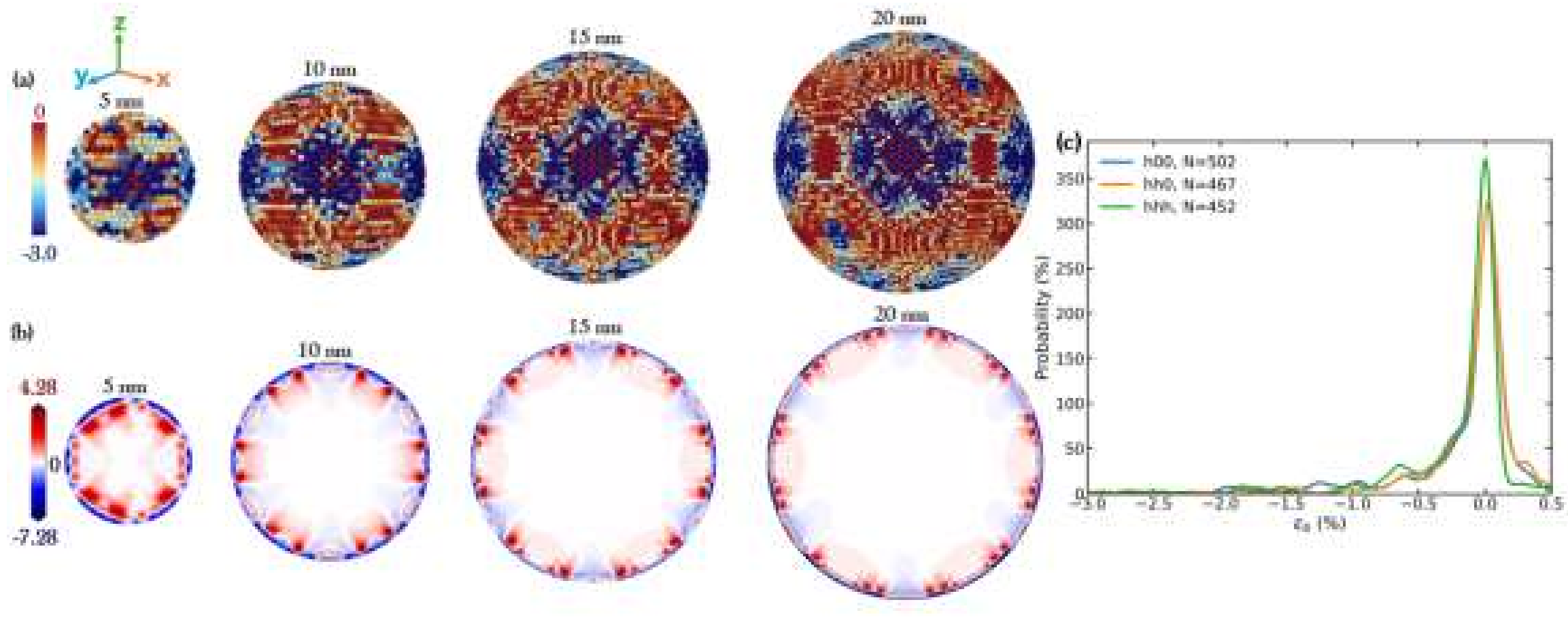

Figure S3: (a) *Surface-projection stress maps for 5, 10, 15 and 20 nm Ti nanospheres and (b) their corresponding cross sections. Compressive stress = blue, tensile stress = red. (c) Average local strain across the three cross sections (hh0) (hh0) and (hhh).*

Supplementary Note 3: Warren plots

The Warren plots (WP) are modelled with the following Fourier space function

$$exp\left[-\frac{q_B^2}{2}\langle\Delta L(L)^2\rangle_{hkl}\right] \quad \text{(S1)}$$

where the WP is proportional to the pair distance with the following relationship for a Gaussian strain model

$$\sqrt{\langle\Delta L(L)^2\rangle}_{hkl} \propto \beta\, L \quad \text{(S2)}$$

where β is the slope of the WP. Due to the complex trends of the WP for the spheres in vacuum, a sixth order polynomial is required to fit the WP, assuming an isotropic model for all the directions in the form:

$$\sqrt{\langle\Delta L(L)^2\rangle} = a0 + a\,L + b\,L^2 + c\,L^3 + d\,L^4 + e\,L^5 + f\,L^6 \quad \text{(S3)}$$

The fits of the Pd WPs in the <h00> family of directions are shown in Figure S4a, with tabulated coefficients given in the table beside the plot. The fitting using this model gives an R_{wp} of 2.404 % with the pattern given in Figure S4b. Performing a similar analysis with equation S2, an R_{wp} of 2.587 % was obtained, meaning that

no significant improvement is observed using the full trends of the WPs. This means that equation S3 is an effective way to model the microstrain of the nanosphere. TOPAS offers the possibility of modelling such contribution by convolving the line profile with Voigt functions, having the same effect as equation S3, however, performing the fit significantly faster. As demonstrated in Figure S4c, the slopes using the Voigt and equation S3 models retrieve identical trends, retrieving the same microstrain values, with a slightly lower R_{wp} of 2.491 %. The Voigt model is therefore preferred for the analysis of the microstrain and will be used henceforth.

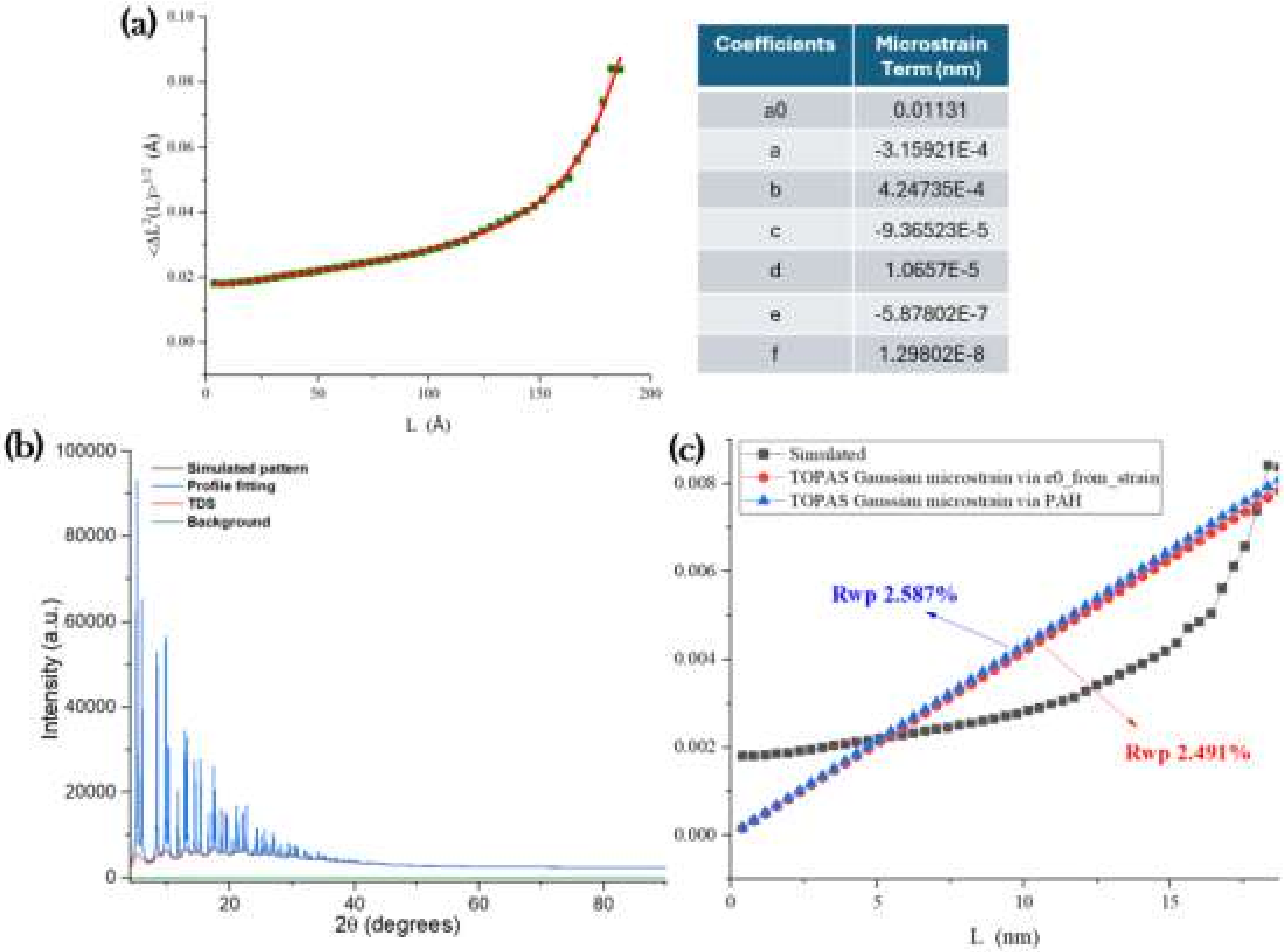

Coefficients	Microstrain Term (nm)
a0	0.01131
a	-3.15921E-4
b	4.24735E-4
c	-9.36523E-5
d	1.0657E-5
e	-5.87802E-7
f	1.29802E-8

Figure S4: (a) Sixth-order polynomial fit to the simulated Pd Warren plot in the <h00> direction; the fitted coefficients are listed in the insert table. (b) Representative diffraction profile fit obtained using the polynomial microstrain distribution. (c) Comparison between the simulated Warren plot (black symbols) and TOPAS Gaussian microstrain models obtained from the polynomial strain representation and from equation S2 (blue symbols). The red symbols correspond to the values obtained using the Voigt approach for microstrain.

As indicated in Section 3.2 of the manuscript, the WPs obtained for Fe and Ti are as shown below in Figure S5.

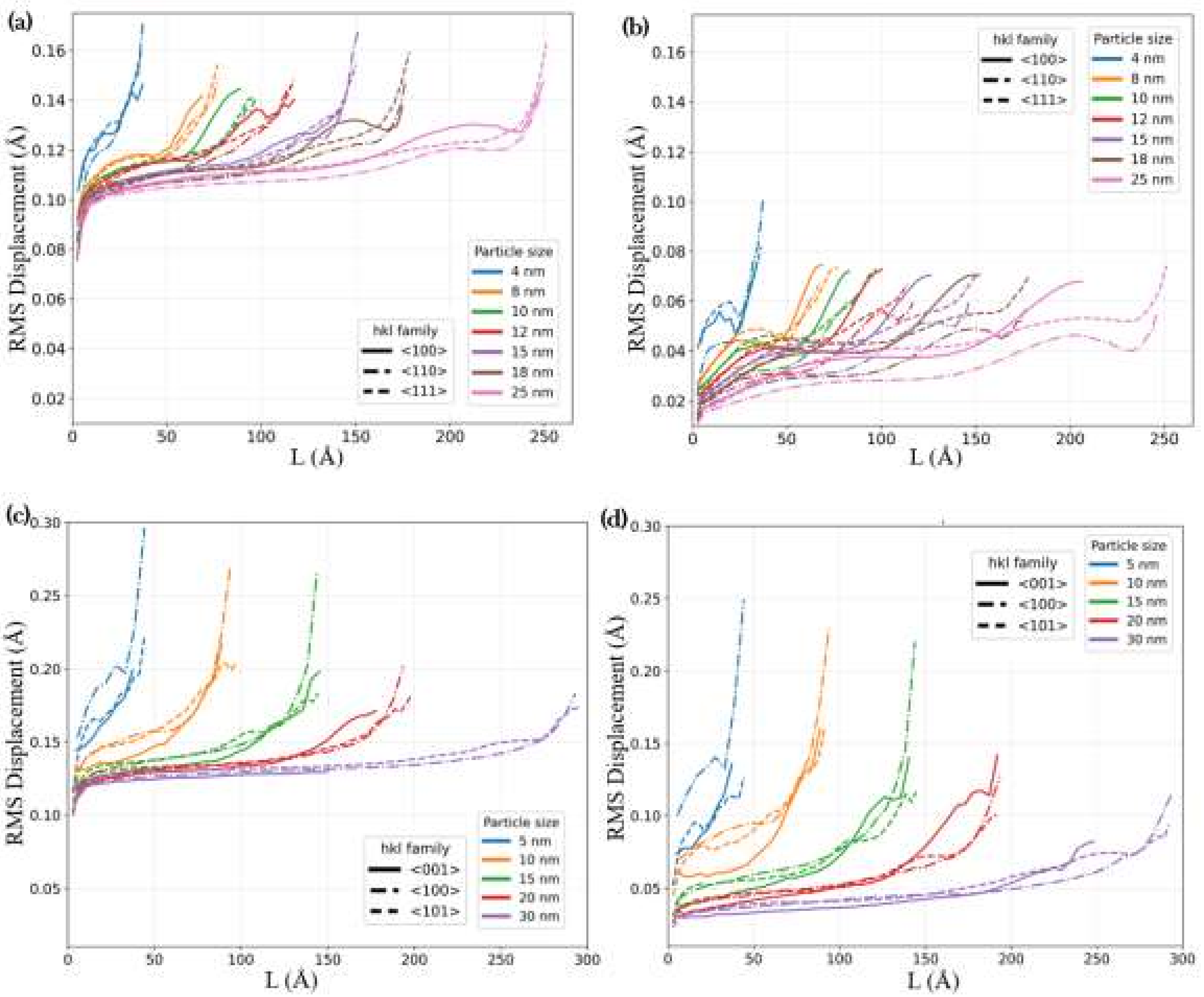

Figure S5: *Warren plots for the Fe (a,b) and Ti (c,d) nanospheres of different size, along the three crystallographic directions, <100>, <110>, <111>. The WPs are obtained from (a), (c) instantaneous coordinates, sampled for 100 frames of the steady-state MD simulation, and (b), (d) time-averaged atomic coordinates.*

Supplementary Note 4: Nanoscale Models

S4.1. Detailed development of the microstrain model

This Supplementary section develops the physical rationale behind the microstrain model introduced in § 4.3 of the main text. In particular, it provides the scaling argument leading to the ideal $1/D^2$ small-particle limit, the alternative shell-confined limit, and the heuristic basis of the mixed fitting form adopted in Eq. (17) of the main manuscript.

We consider the local strain field inside a nanocrystal as

$$\varepsilon(r,D) = \bar{\varepsilon}(D) + \delta\varepsilon(r,D) \qquad \text{(S4)}$$

where $\bar{\varepsilon}(D)$ is the volume-averaged strain and $\delta\varepsilon(r,D)$ is the local fluctuation around that average. The effective microstrain measured by diffraction line-profile analysis is identified with the root-mean-square amplitude of the fluctuating part of the field,

$$\varepsilon_{micro}(r,D) = \sim\sqrt{\langle[\delta\varepsilon(\boldsymbol{r},D)]^2\rangle} = \left[\frac{1}{V}\int_V \left(\delta\varepsilon(\boldsymbol{r},D)\right)^2 dV\right]^{1/2} \qquad \text{(S5)}$$

where $V \propto D^3$ is the nanocrystal volume. In the present model, $\delta\varepsilon$ originates from the non-uniform surface stress field. Let $\tau_{surf}(s, D)$ denote the local surface stress along the nanocrystal boundary, where s is a coordinate along the surface. We further define:

- $\sigma_0(D)$: characteristic internal stress induced by the surface boundary conditions;
- $\Delta\tau_{surf}$: typical variation in surface stress between different facets, edges or surface.

Schematically represented in Figure S6. If the surface were perfectly uniform, only the average surface contribution would remain, producing an average internal stress and a corresponding mean lattice strain, but no microstrain. In real nanocrystals, however, the surface is not uniform because of faceting, edges, vertices, local curvature differences, and variations in atomic coordination. It is therefore natural, at the level of scaling laws, to assume that the characteristic variation of the local surface stress is of the same order as the characteristic surface-induced internal stress scale:

$$\Delta\tau_{surf} \sim \sigma_0(D) \tag{S6}$$

For a nearly spherical nanocrystal, the latter follows the same capillarity-controlled size dependence as the average internal compressive stress, i.e.

$$\sigma_0(D) \sim 1/D \tag{S7}$$

For small, strongly faceted nanocrystals, the characteristic lateral distance between surface regions with distinct surface stress is of order *D*. The corresponding gradient along the surface then scales as

$$\frac{\partial\tau_{surf}}{\partial s} \sim \frac{\Delta\tau_{surf}}{\Delta s} \sim \frac{\sigma_0(D)}{D} \sim \frac{1}{D^2} \tag{S8}$$

Thus, surface stress *heterogeneity* produces a gradient of order $1/D^2$.

S4.1.1. Coarse-graining and local strain fluctuations

The surface-stress gradient alone is not yet a bulk microstrain. To define a local bulk quantity, one performs coarse-graining over a region of microscopic radius *l*, typically of the order of a few Å (Figure S7). The local stress fluctuation in such a region is then estimated as

$$\delta\sigma_{local} \sim \frac{\partial\tau_{surf}}{\partial s}\, l \sim \frac{1}{D^2} l \tag{S9}$$

The key point is that *l* is a microscopic averaging length and does not scale with the nanocrystal diameter *D*. Therefore eq. (S9) immediately implies

$$\delta\sigma_{local} \sim \frac{1}{D^2} \tag{S10}$$

Through linear elasticity, the corresponding local strain fluctuation scales as

$$\delta\varepsilon_{local} \sim \frac{\delta\sigma_{local}}{E_{eff}} \sim \frac{1}{D^2} \tag{S11}$$

where E_{eff} is an effective elastic modulus in the relevant crystallographic direction. Elastic anisotropy is not treated explicitly in this averaged discussion because it mainly affects the prefactor, not the scaling exponent.

If the field of strain fluctuations extends over the entire nanocrystal volume, or at least over a substantial fraction of it, then eq. (S5) gives

$$\varepsilon_{micro}(D) \sim \left[\frac{1}{V}\int_V \left(\delta\varepsilon(\boldsymbol{r}, D)\right)^2 dV\right]^{1/2} \sim \frac{1}{D^2} \tag{S12}$$

This is the natural leading-order law for small, strongly faceted nanocrystals. It is the quantity identified with the effective microstrain extracted from diffraction line-profile analysis, including the constant-microstrain treatment used in TOPAS.

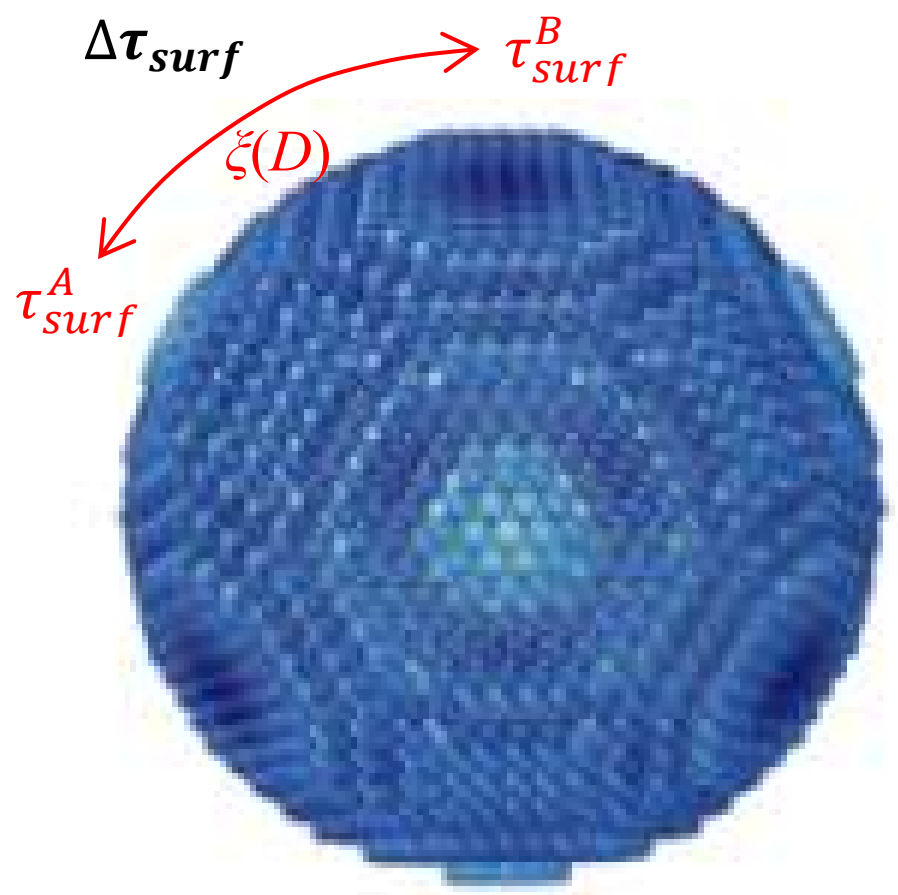

Figure S6: *Schematic representation of surface-stress heterogeneity on a faceted nanocrystal surface, adapted from the stress-map evidence of Figure 1 in the main text.* τ^A_{surf} *and* τ^B_{surf} *denote local surface-stress states on different surface patches, while* $\boldsymbol{\Delta\tau_{surf}}$ *is their characteristic difference. The symbol ξ(D) indicates the characteristic lateral distance over which the surface stress changes appreciably.*

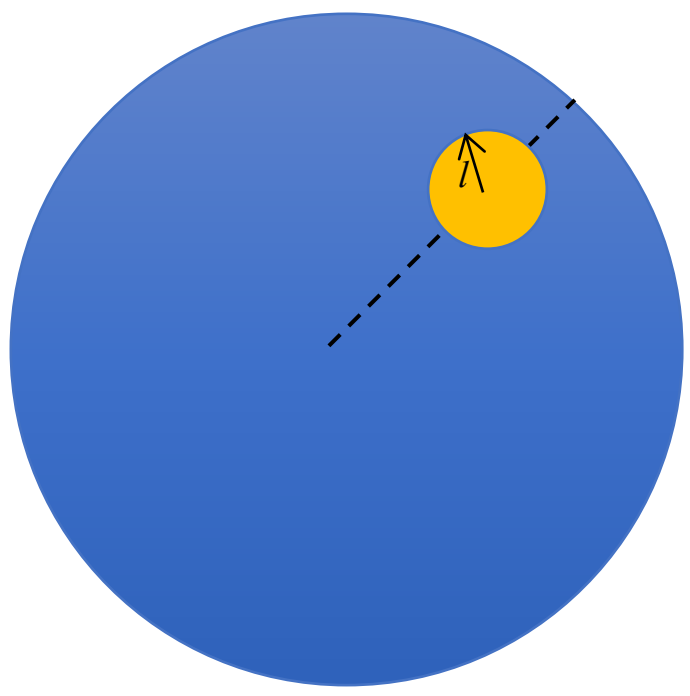

Figure S7: ***Schematic representation of local coarse-graining in the near-surface region. The large sphere represents the nanocrystal, while the small sphere represents the local averaging volume of radius*** *l used to define a coarse-grained bulk quantity such as local stress or local strain. Although* $\tau_{surf}(s, D)$ *is defined on the particle boundary, its spatial non-uniformity induces an inhomogeneous elastic field inside the nanocrystal.*

S4.1.2. Alternative shell-confined limit

It is useful to consider an alternative limiting case. If the local strain fluctuations $\delta\varepsilon_{local} \sim 1/D^2$ were confined to a thin outer shell of thickness *t* independent of *D*, the affected volume fraction would scale as

$$\frac{V_{shell}}{V} \sim \frac{t\,D^2}{D^3} \sim \frac{1}{D} \tag{S13}$$

The square microstrain would then scale as

$$\varepsilon^2_{micro}(D) \sim \frac{V_{shell}}{V} \times (\delta\varepsilon_{local})^2 \sim \frac{1}{D} \times \frac{1}{D^4} = \frac{1}{D^5} \tag{S14}$$

that is

$$\varepsilon_{micro}(D) \sim \frac{1}{D^{5/2}} \quad \text{(S15)}$$

This is a significantly faster decay than the $1/D^2$ behaviour discussed above. The fact that the latter is the relevant leading-order trend indicates that the inhomogeneous strain field is not simply confined to a thin surface shell, but extends over a substantial fraction of the nanoparticle volume, at least in the small-size regime considered here. This interpretation is also consistent with the MD evidence discussed in the main text.

S4.2. Heuristic crossover model based on a size-dependent correlation length

The $1/D^2$ law should be regarded as the ideal small-particle limit, appropriate when the characteristic lateral distance over which the surface stress changes is of order *D*. As particle size increases and the morphology becomes smoother, surface-stress variations may become more localized, and the associated strain field may occupy a smaller or less uniformly weighted fraction of the volume. This motivates a heuristic refinement of the model.

Let $\xi(D)$ denote the characteristic lateral distance over the surface over which the surface stress changes appreciably. The characteristic internal stress scale still behaves as $\sigma_0(D) \sim 1/D$, but the corresponding surface-stress gradient is now written more generally as

$$\frac{\partial \tau_{surf}}{\partial s} \sim \frac{\sigma_0(D)}{\xi(D)} \sim \frac{1}{D\,\xi(D)} \quad \text{(S16)}$$

For a coarse-grained region of fixed microscopic size *l*, the resulting local stress and strain fluctuations scale as

$$\delta\sigma_{local} \sim \frac{l}{D\,\xi(D)} \quad \text{(S17)}$$

$$\delta\varepsilon_{local}(D) \sim \frac{1}{D\,\xi(D)} \quad \text{(S18)}$$

A second assumption is required concerning the penetration depth of a surface-stress modulation into the crystal. In linear elasticity, a boundary modulation of lateral wavelength ξ typically decays over a depth of the same order. At the level of scaling laws, it is therefore reasonable to take the affected volume fraction as

$$f(D) \sim \frac{\xi(D)}{D} \quad \text{(S19)}$$

The volume-averaged RMS microstrain then scales as

$$\varepsilon_{micro}(D) \sim \sqrt{f(D)\,\langle[\delta\varepsilon_{local}]^2\rangle} \sim \sqrt{1/[\xi(D)\,D^3]} \quad \text{(S20)}$$

This expression reduces to the previously derived $1/D^2$ law when $\xi(D) \sim D$, as expected for small nanocrystals with global faceting, but tends to a $1/D^{3/2}$ law if the correlation length is weakly dependent on or independent of *D*. A convenient interpolating choice is

$$\xi(D) = \frac{D\,D_c}{D + D_c} \quad \text{(S21)}$$

where D_c is the crossover. Substituting eq. (S21) into eq. (S20) gives

$$\varepsilon^{c}_{micro}(D) \sim \frac{k_c}{D^2}\sqrt{1 + \frac{D}{D_c}} \quad \text{(S22)}$$

so that $\varepsilon^{c}_{micro}(D) \sim 1/D^2$ for $D << D_c$, while $\varepsilon^{c}_{micro}(D) \sim 1/D^{3/2}$ for $D >> D_c$. This crossover form is heuristic, because it relies on the specific assumption that the penetration depth of the elastic inhomogeneity remains of order $\xi(D)$. It is physically suggestive, but not necessarily general.

S4.3. Practical mixed fitting form

If the diffraction-derived microstrain shows a shallower large-*D* tail, or if profile fitting introduces a weak systematic floor, a more pragmatic and often more effective representation is

$$\varepsilon_{micro}(D) = \varepsilon_0 + \frac{C_1}{D} + \frac{C_2}{D^2} \qquad \text{(S23)}$$

This is the expression retained in the main text. It preserves the physically expected $1/D^2$ contribution in the strongly surface-dominated small-size regime, while allowing a weaker $1/D$ correction over larger size ranges. In this interpretation, C_2/D^2 captures the small-particle limit associated with global surface-stress heterogeneity, whereas C_1/D represents an effective large-*D* correction associated with smoother morphology, more localized surface-stress variations, partial confinement of the strain field, or weak systematic contributions from the diffraction analysis itself. The constant term ε_0, if small, may represent a residual size-independent baseline.

Supplementary Note 5

The values of the fitting constants in equations proposed in Section 4 of the manuscript are reported for each dataset corresponding to the fits shown in Figure 5. Table S1 summarizes the fitting parameters for the lattice parameter analysis, Table S2 for the Debye-Waller coefficient analysis and Table S3 for the microstrain analysis.

Table S1: Summarizes the fitting parameters obtained from the lattice parameter analysis of the simulated nanoparticle systems.

System	Class	Structure	a_∞ (Å)	A_a (Å.nm)	A_a / a_∞(%)	R^2	Plot
Pd NPs (spheres)	Metal	FCC	3.909179(80)	0.08336(88)	2.13	0.993	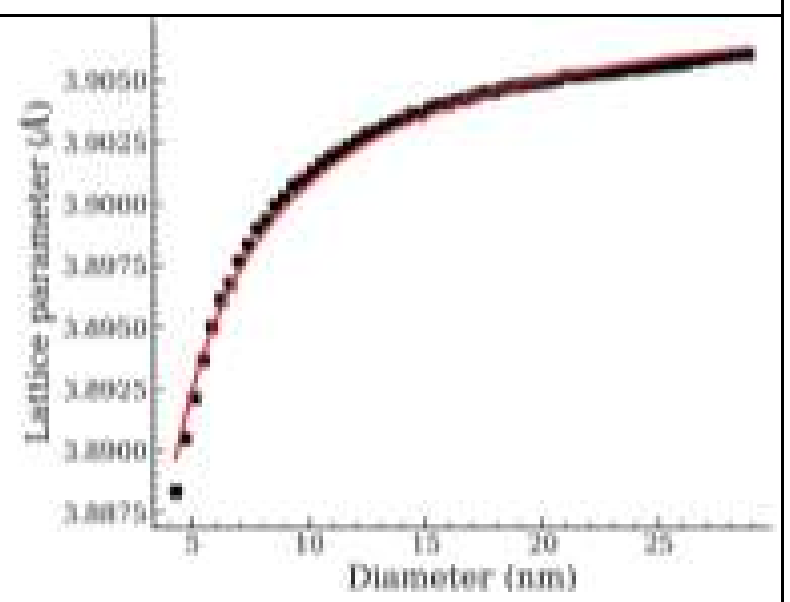
Fe NPs (spheres)	Metal	BCC	2.859167(21)	0.02564(23)	0.89	0.993	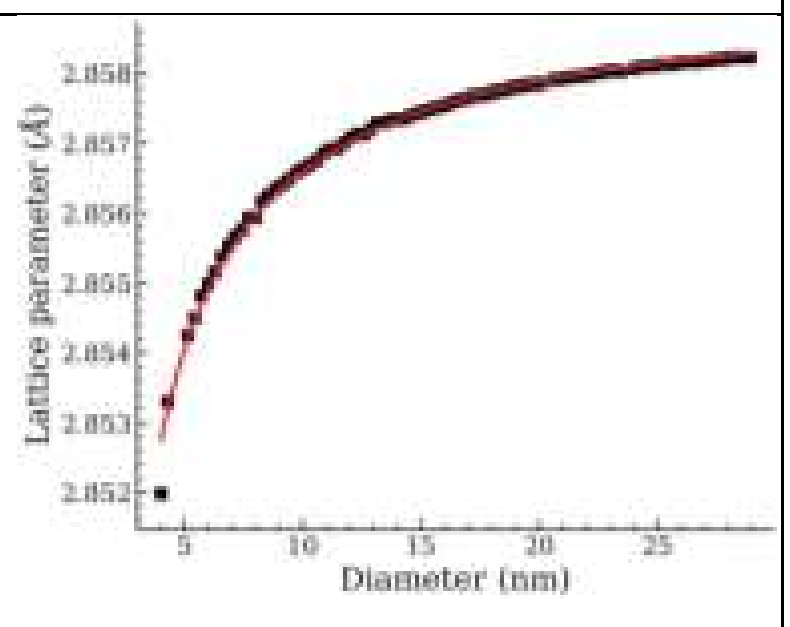

Ti NPs (spheres)	Metal	HCP(a axis)	2.95004(18)	0.0393(19)	1.33	0.988	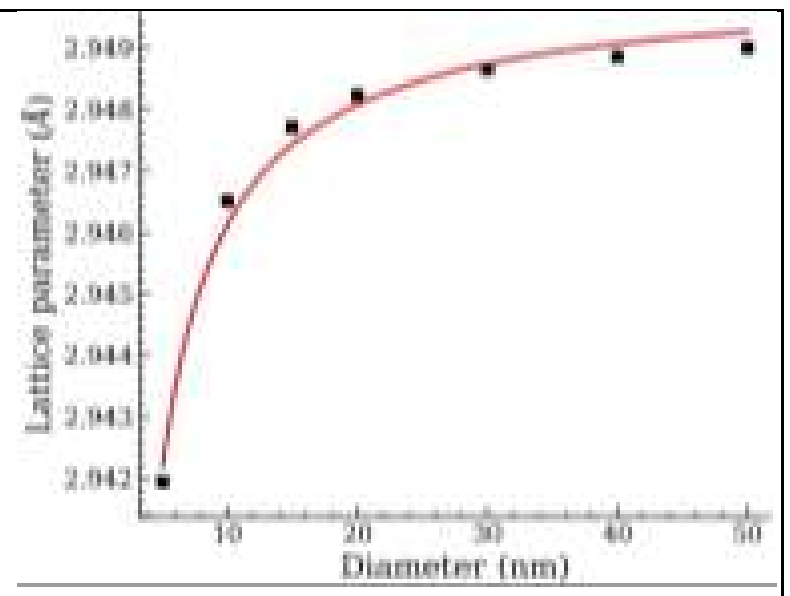
		HCP (c axis)	4.70564(24)	0.0330(26)	0.70	0.970	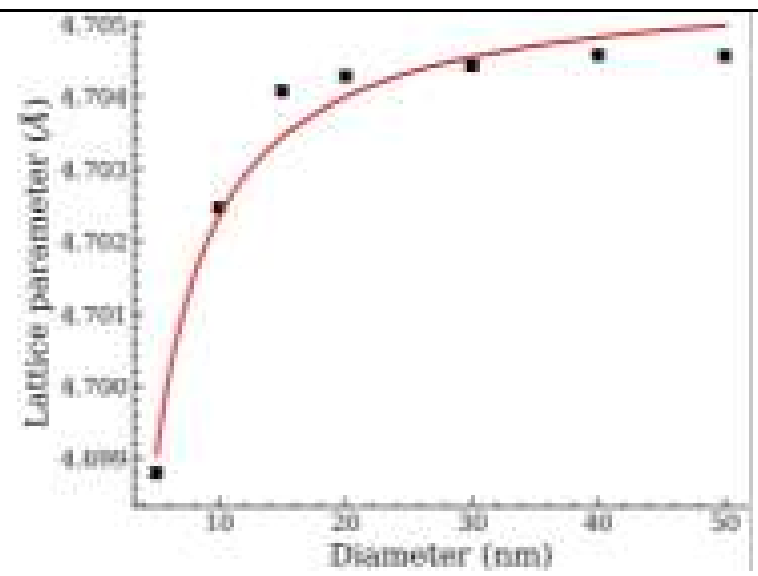

Table S2: Summarizes the fitting parameters obtained from Debye-Waller coefficient analysis of the simulated nanoparticle systems.

**System**	**Class**	**Structure**	B_∞ **(Å^2)**	C_B **(Å^2.nm)**	**R^2**	**Plot**
Pd NPs (simulated)	Metal	FCC	0.53241(38)	0.1266(42)	0.935	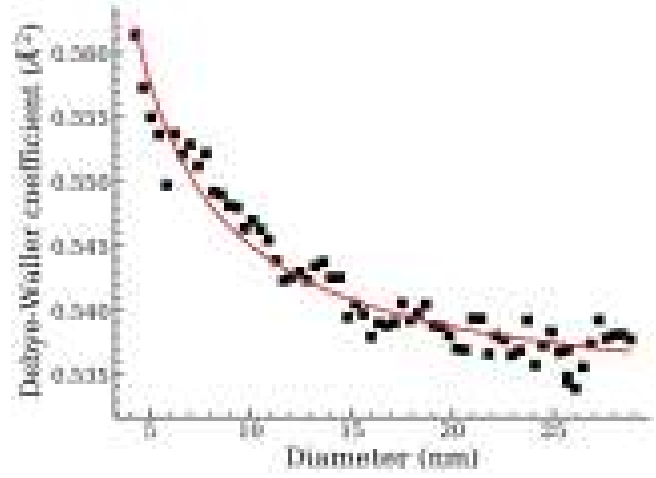
Fe NPs (simulated)	Metal	BCC	0.38414(53)	0.4186(58)	0.984	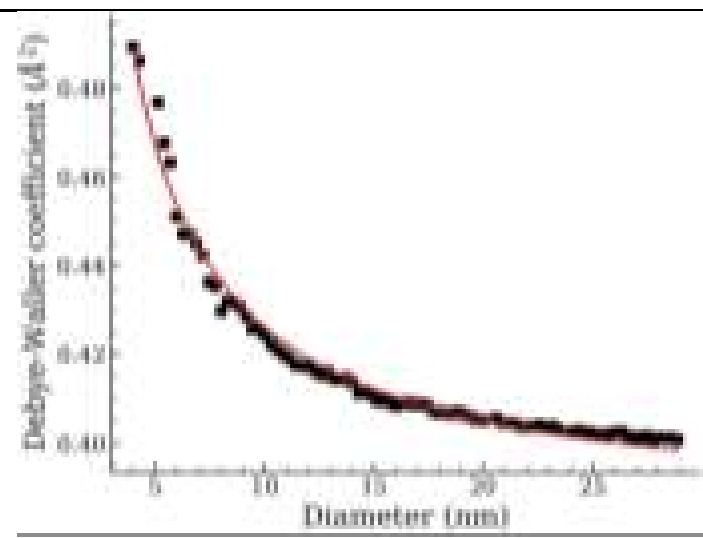
Ti NPs (simulated)	Metal	HCP	0.5599(36)	1.007(39)	0.993	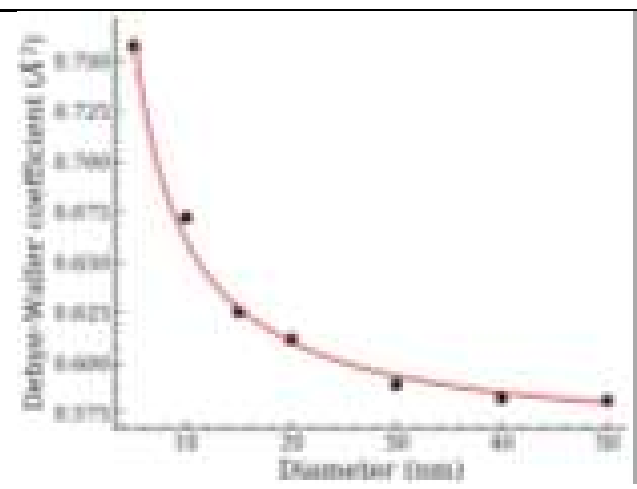

Table S3: Summarizes the fitting parameters obtained from the microstrain analysis of the simulated nanoparticle systems.

System	Class	Structure	ε_∞	C_1	C_2	R^2	Plot
Pd NPs (spheres)	Metal	FCC	2.3(43)e-05	4.80(93)e-03	4.87(39)e-02	0.990	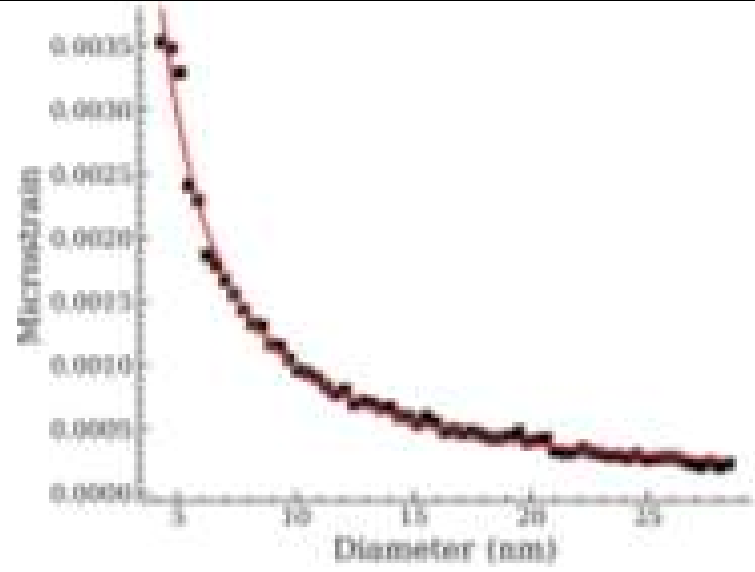
Fe NPs (spheres)	Metal	BCC	1.56(32)e-04	3.33(51)e-03	2.51(13)e-02	0.989	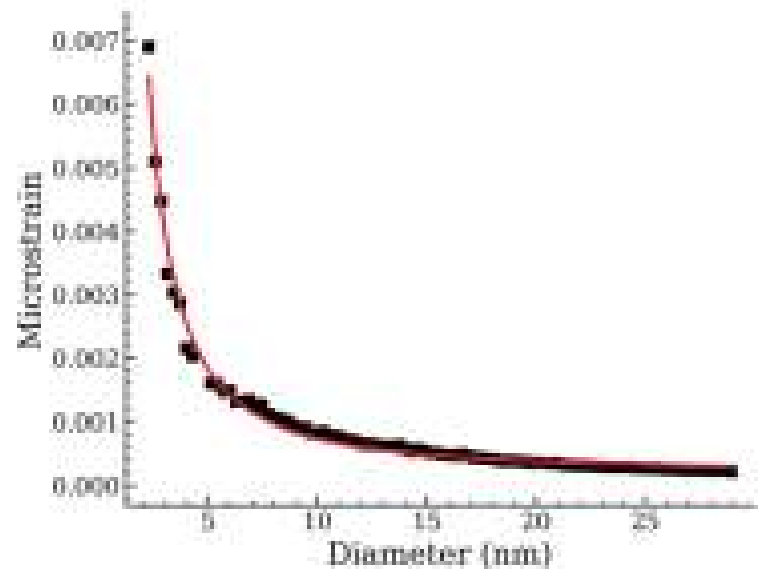
Ti NPs (spheres)	Metal	HCP	8.2(57)e-05	4.5(15)e-03	3.91(67)e-02	0.998	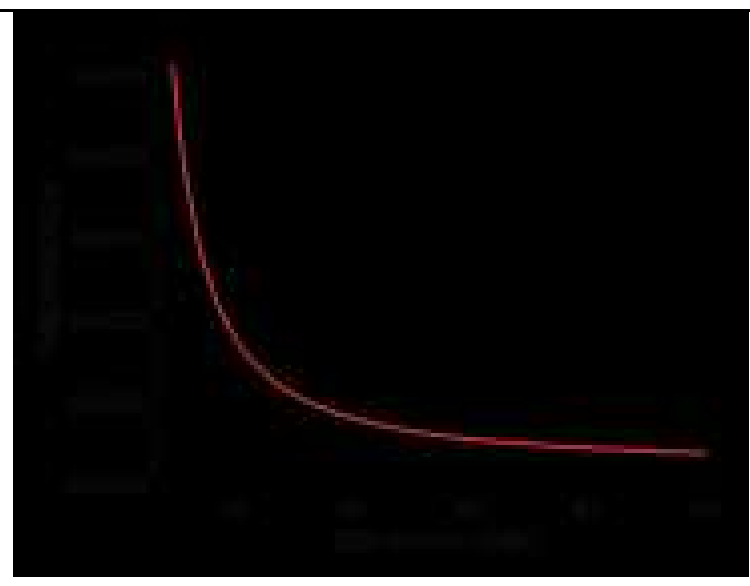

Literature Case studies

For all the case studies discussed in section 5.2 of the manuscript, tables S4-S6 summarize the fitting constants extracted from the nanoscale model fits.

Table S4: Summary of the fitting parameters obtained from the lattice parameter analysis of different literature case studies.

System	Class	Structure	a_∞ (Å)	A_a (Å.nm)	A_a/a_∞(%)	R^2	Plot	Trend
Silver NPs (cube) (Chen *et al.*, 2017)	Metal	FCC	4.08991(32)	0.1553(10)	3.79	0.999	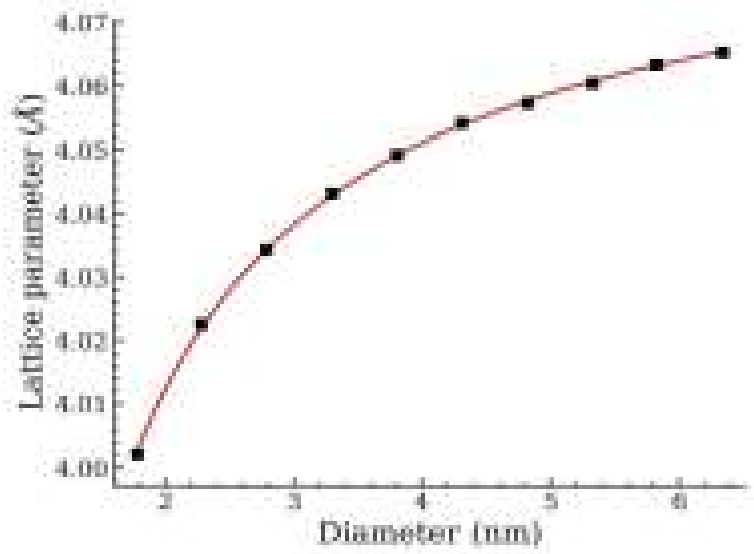	Contraction

Silver NPs (cuboctahedron) (Chen *et al.*, 2017)	Metal	FCC	4.09174(46)	0.1429(14)	3.49	0.999	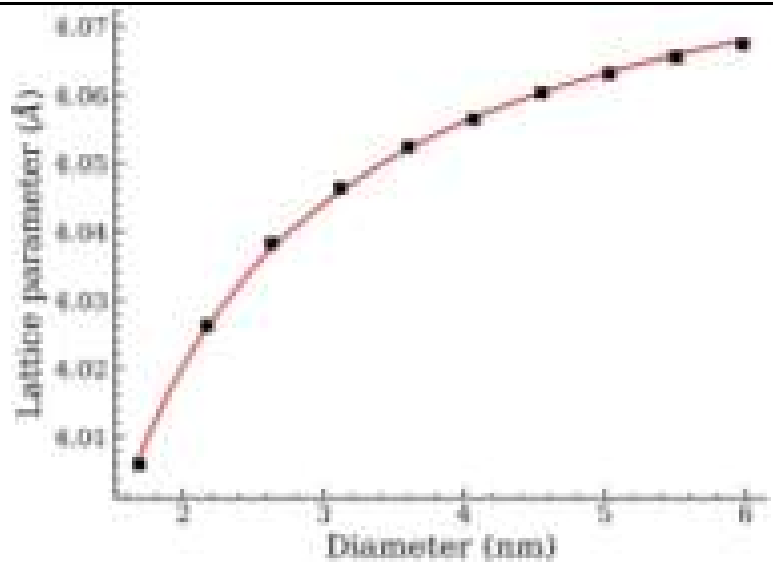	Contraction
Silver NPs (octahedron) (Chen *et al.*, 2017)	Metal	FCC	4.09137(25)	0.13263(75)	3.24	0.999	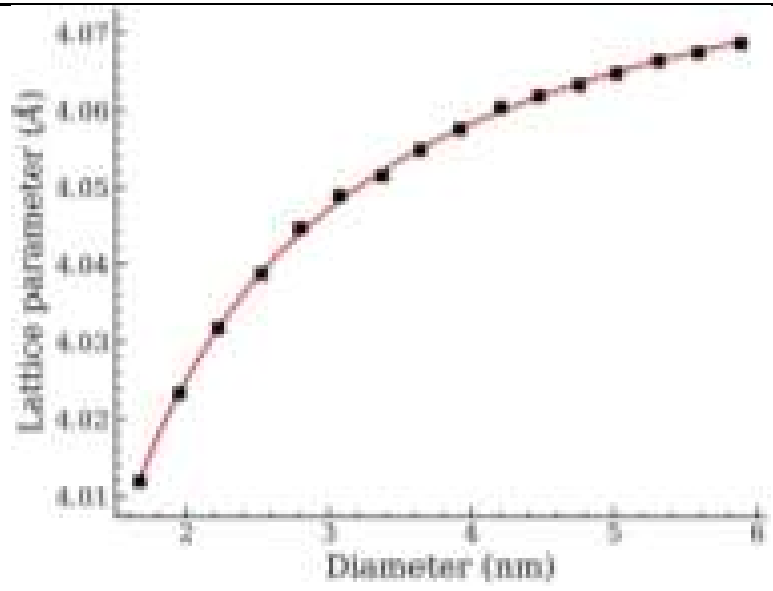	Contraction
Carbon supported Pt NPs (Wulff polyhedral) (Leontyev *et al.*, 2014)	Metal/support	FCC	3.9254(14)	0.0717(56)	1.82	0.852	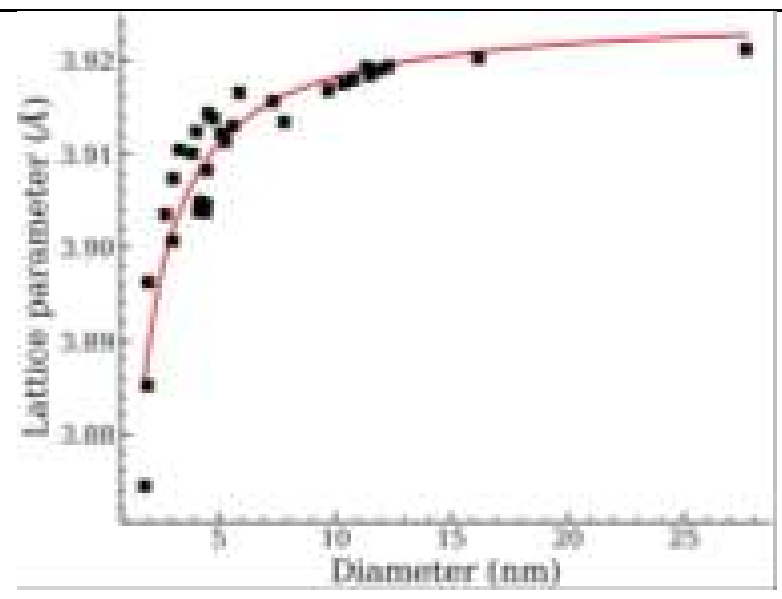	Contraction
CeO_2 NPs (spherical) (Subbareddy *et*	Metal oxide	FCC	5.4342(20)	0.0629(81)	1.15	0.895	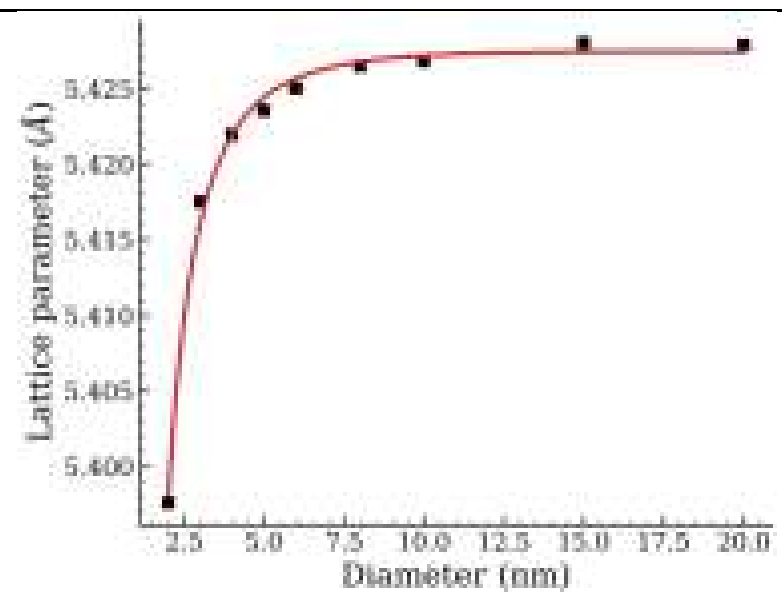	Contraction

al., 2026)			5.42682 (77)	- 0.0081(32)	-0.14	0.476	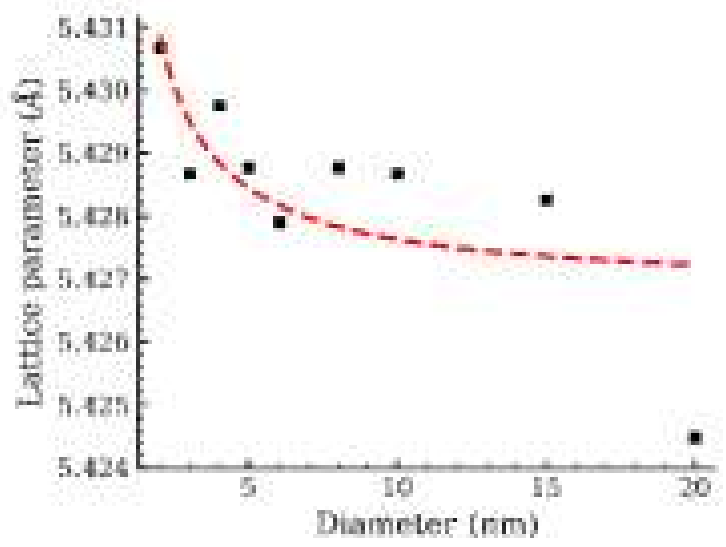	Expansion
CeO_2 NPs (polyhedral) (Prieur *et al.*, 2020)	Metal oxide	FCC	5.4068(18)	- 0.0858(91)	-1.58	0.928	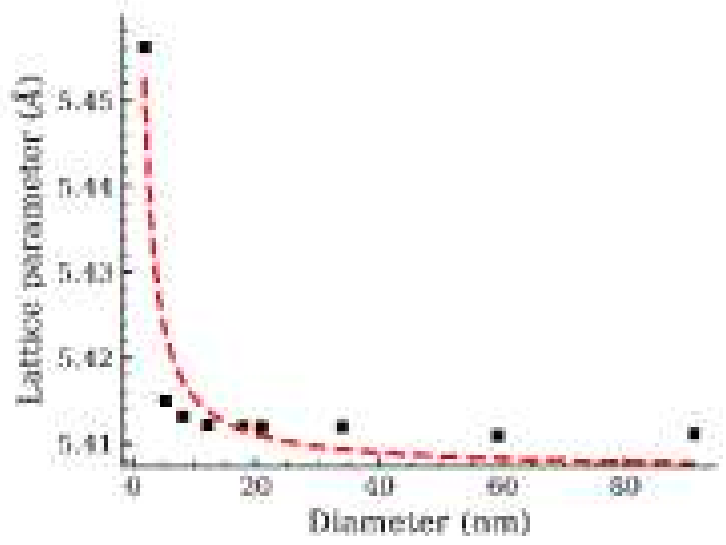	Expansion
MgO NPs (polyhedral) (Barad *et al.*, 2024)	Metal oxide	FCC	4.21075 (47)	- 0.235(2 1)	-5.58	0.970	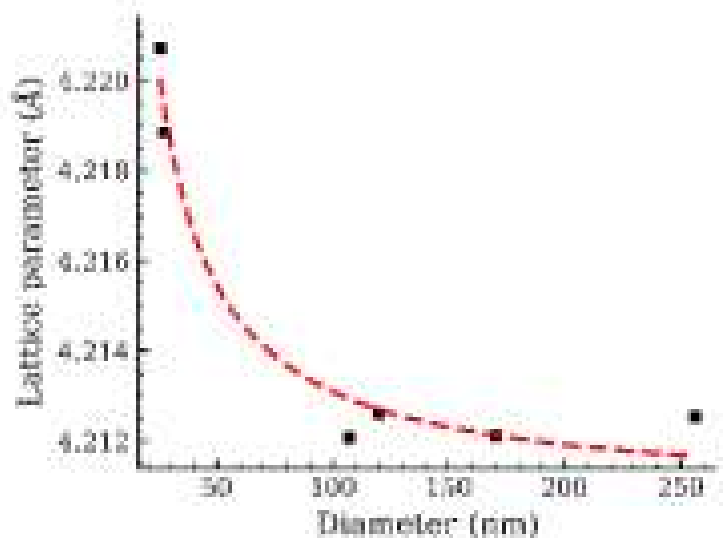	Expansion
NiCuZn (granular) (He *et al.*, 2015)	Transition metal oxide	Fd-3m	8.4121(98)	- 0.0045(18)	-0.05	0.614	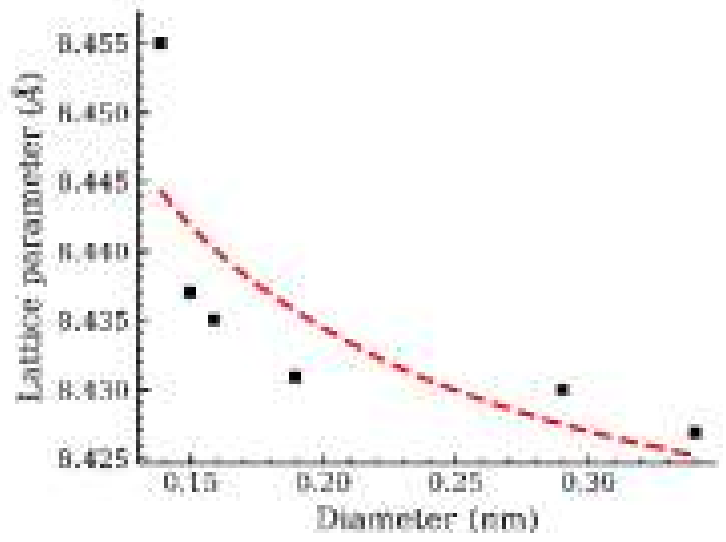	Expansion

Table S5: Summary of the fitting parameters obtained from the Debye-Waller coefficient analysis of different literature case studies.

**System**	**Class**	**Structure**	B_0 **(Å²)**	C_B **(Å².nm)**	**R²**	**Plot**
Ag NPs (model derived) (Sadaiyandi, 2009)	Metal	FCC	2.045(26)	3.10(15)	0.978	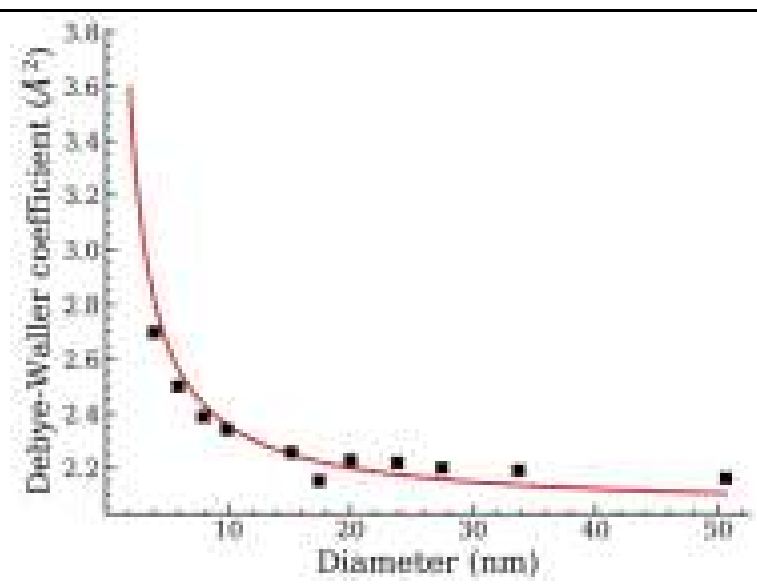

Al NPs (model derived) (Sadaiyandi, 2009)	Metal	FCC	2.556(69)	6.01(37)	0.966	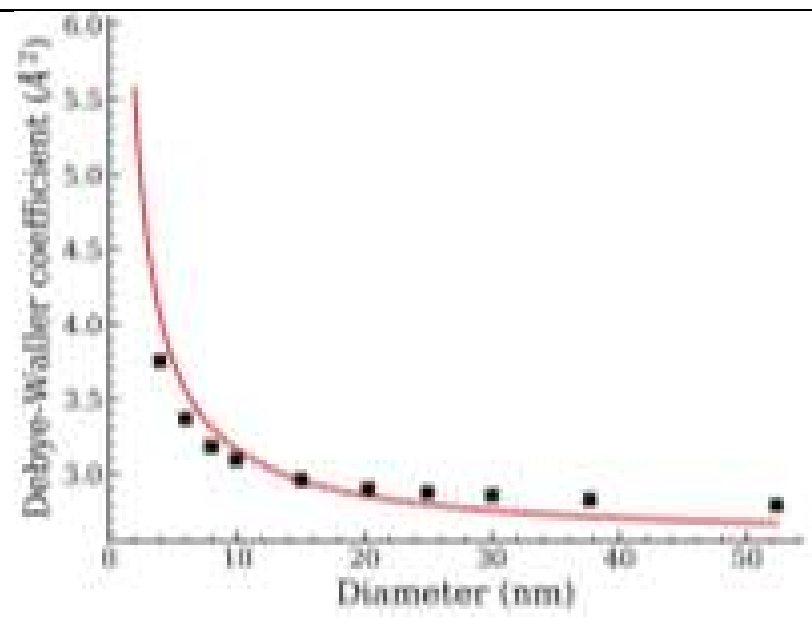
Au NPs (simulated) (Batyrow *et al.*, 2023)	Metal	FCC	0.7133(29)	0.808(24)	0.997	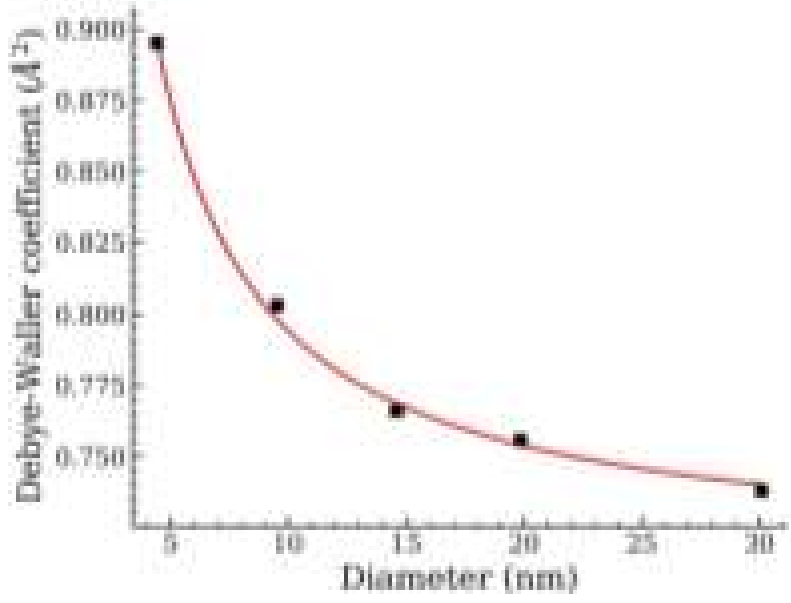
CeO_2 NPs (simulated) (Subbareddy *et al.*, 2026)	Metal oxide	FCC	0.1572(95)	1.165(89)	0.988	Ce 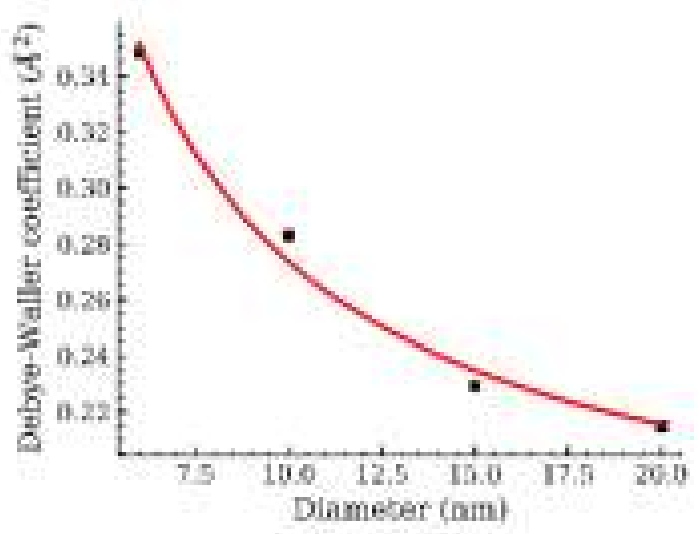
			0.301(46)	1.55(43)	0.867	O 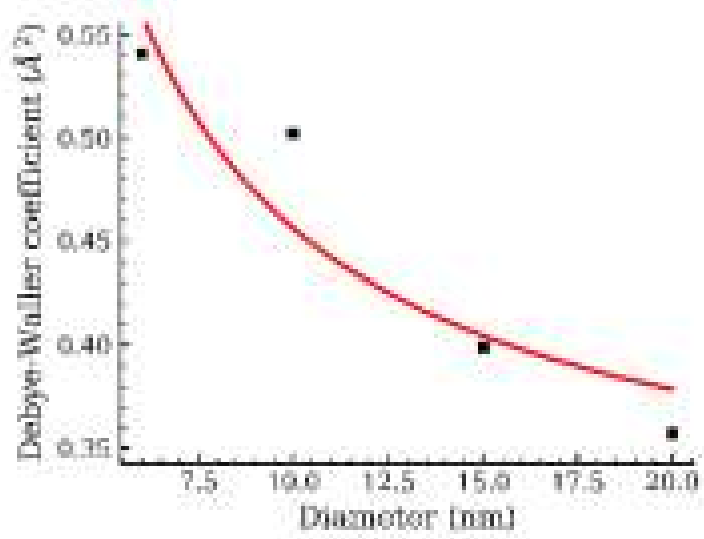

Table S6: Summary of the fitting parameters obtained from the microstrain analysis of different literature case studies.

**System**	**Class**	ε_∞	C_1	C_2	R^2	**Plot**
ZnO (Prabakar & Muthukumaran, 2021)	Metal oxide	1.40(100)e-03	3.01(31)e-02	3.9(24)e-03	0.999	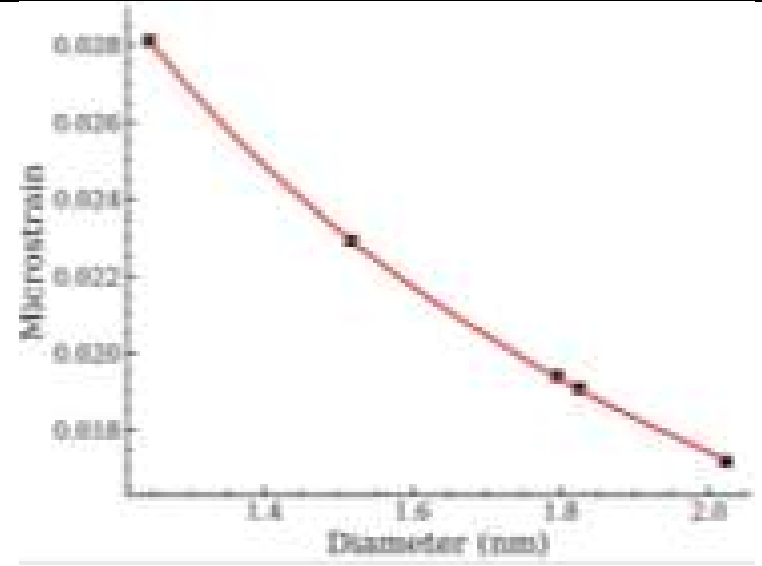

CeO_2 (Prieur *et al.*, 2020)	Metal oxide	9.2(38)e-04	7.73(67)e-02	5.8(16)e-02	0.998	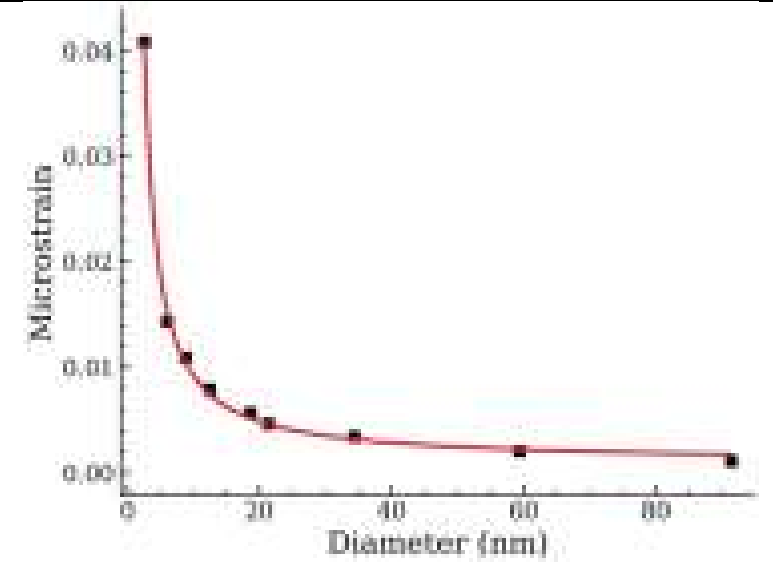
WO_3 (Salim *et al.*, 2023)	Metal oxide	1.16(54)e-03	8(21)e-03	2.7(20)e-01	0.983	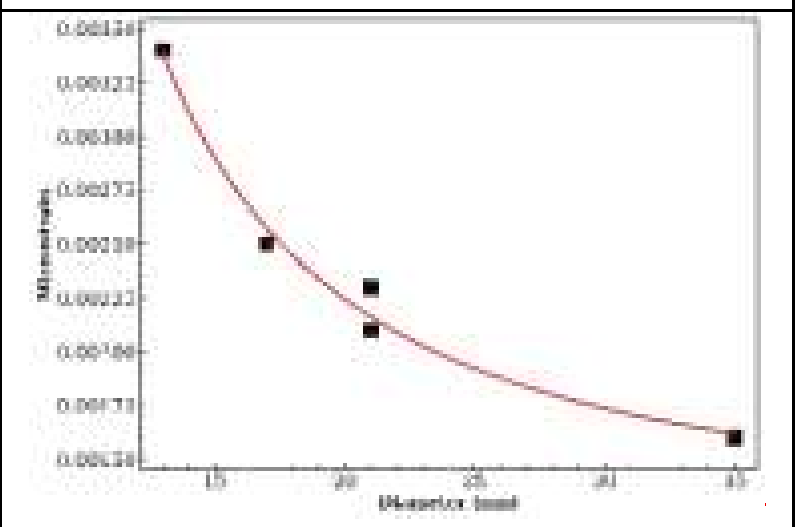
Cu-Ni-W (Saini *et al.*, 2025)	Metal alloy	4.53(41)e-03	3.9(57)e-03	7(149)e-04	0.783	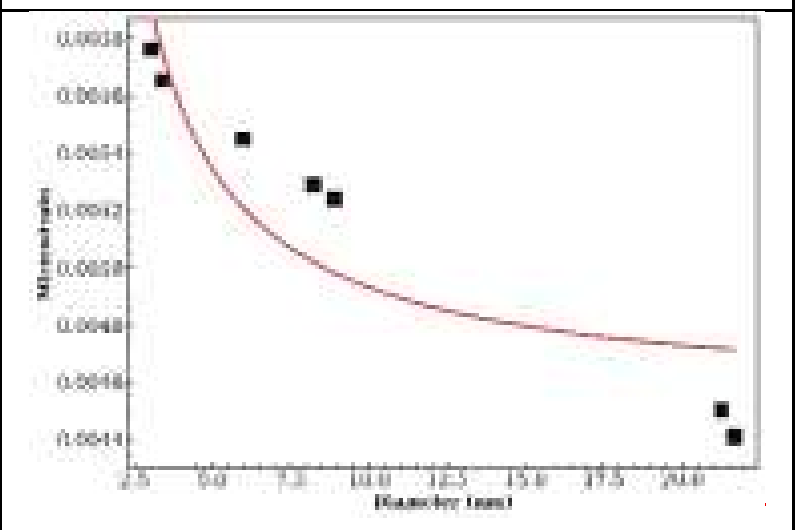

The plots here represent a decrease in the microstrain with increasing size of the nanoparticles. The source of microstrain is different in each of the case, there are factors of dislocation density, doping species and surface coordination effects. The authors infer microstrain from diffraction peak broadening and do not directly resolve the distribution of strain on the surface. In particular, the role of heterogeneous surface environments arising from facets, edges, corners or the variation in the local atomic coordination is not explicitly addressed.

References for SI

Batyrow, M., Erucar, I. & Öztürk, H. (2023). *Concurr. Comput. Pract. Exp.* **35**, e7566.
Barad, C., Kimmel, G., Opalińska, A., Gierlotka, S. & Łojkowski, W. (2024). *Heliyon.* **10,** e31275.
Chen, L., Wang, Q. & Xiong, L. (2017). *J. Nanoparticle Res.* **19**, 300.
Cullity, B. D. (1978). *Answers to problems: elements of X-ray diffraction.* Addison-Wesley Publishing Company.
Guinier, A. & Fournet, G. (1955). *Small-angle Scattering of X-rays.* John Wiley & Sons, Inc.
He, X.-M., Yan, S.-M., Li, Z.-W., Zhang, X., Song, X.-Y., Qiao, W., Zhong, W. & Du, Y.-W. (2015). *Chinese Phys. B.* **24**, 127502.
Leontyev, I. N., Kuriganova, A. B., Leontyev, N. G., Hennet, L., Rakhmatullin, A., Smirnova, N. V. & Dmitriev, V. (2014). *RSC Adv.* **4**, 35959–35965.
Prabakar, C. & Muthukumaran, S. (2021). *J. Mater. Sci. Mater. Electron.* **32**, 9702–9720.
Prieur, D., Bonani, W., Popa, K., Walter, O., Kriegsman, K. W., Engelhard, M. H., Guo, X., Eloirdi, R., Gouder, T., Beck, A., Vitova, T., Scheinost, A. C., Kvashnina, K. & Martin, P. (2020). *Inorg. Chem.* **59**, 5760–5767.
Sadaiyandi, K. (2009). *Mater. Chem. Phys.* **115**, 703–706.
Saini, H. & Khatri, M. S. (2025). *Bull. Mater. Sci.* **48,** 95.
Sakuma, T. (1995). *Bull. Electrochem.* **11**, 57–80.
Salim, E. T., Hassan, A. I., Mohamed, F. A., Fakhri, M. A. & Addie, A. J. (2023). *J. Mater. Sci. Mater. Electron.* **34**, 1546.

Scardi, P., Ricardo, C. L. A., Perez-Demydenko, C. & Coelho, A. A. (2018). *J. Appl. Crystallogr.* **51**, 1752–1765.
Scardi, P., Leonardi, A., Gelisio, L., Suchomel, M. R., Sneed, B. T., Sheehan, M. K. & Tsung, C. K. (2015). *Phys. Rev. B.* **91**, 155414.
Scardi, P., Leoni, M. & Delhez, R. (2004). *J. Appl. Crystallogr.* **37**, 381–390.
Scardi, P. & Malagutti, M. A. (2024). *Cryst. Growth Des.* **24**, 4380–4392.
Subbareddy, S., Malagutti, M. A., Nautiyal, H., Ataollahi, N. & Scardi, P. (2026). *Nanomaterials* **16**, 333.
Tsunekawa, S., Ishikawa, K., Li, Z.-Q., Kawazoe, Y. & Kasuya, A. (2000). *Phys. Rev. Lett.* **85**, 3440–3443.
Wilson, A. J. C. (1949). *Acta Crystallogr.* **2**, 220–222.
